# Quantitative Assessment of Grapevine Wood Colonization by the Dieback Fungus *Eutypa lata*

**DOI:** 10.3390/jof3020021

**Published:** 2017-05-06

**Authors:** Cédric Moisy, Gilles Berger, Timothée Flutre, Loïc Le Cunff, Jean-Pierre Péros

**Affiliations:** 1 Institut Français de la Vigne et du Vin, UMT Géno-Vigne, F-34060 Montpellier, France; loic.lecunff@vignevin.com; 2 INRA, UMR Amélioration Génétique et Adaptation des Plantes méditerranéennes et tropicales, F-34060 Montpellier, France; bergerg@supagro.inra.fr (G.B.); timothee.flutre@inra.fr (T.F.); jean-pierre.peros@inra.fr (J.-P.P.)

**Keywords:** fungus, pathogenicity, susceptibility, real-time PCR, *Vitis vinifera*, *Eutypa lata*, wood colonization

## Abstract

*Eutypa lata* is a fungal pathogen causing severe dieback in vineyards worldwide. This fungus colonizes vines through pruning wounds, eventually causing a brown sectorial necrosis in wood as well as stunted vegetative growth. Several years may pass between infection and the expression of external symptoms, hindering the rapid evaluation of both grapevine cultivars susceptibility and *E. lata* variation in aggressiveness. We aimed to develop a rapid quantitative method for the assessment of wood colonization after inoculation of cuttings in controlled conditions. We used several grape cultivars varying in susceptibility in the vineyard and fungal isolates with different levels of aggressiveness to monitor wood colonization during a maximum period of 2 months. Re-isolation allowed demonstration of the effects of both cultivars and fungal isolates on the rate of wood colonization. We also developed a real-time PCR method that was efficient in measuring fungal biomass, which was found to be correlated with isolate aggressiveness based on foliar symptom severity. The real-time PCR approach appears to be a useful technology to evaluate grapevine susceptibility to *E. lata*, and could be adapted to other pathogens associated with grapevine trunk diseases.

## 1. Introduction

Grapevine trunk diseases (GTD: Eutypa, Esca disease, and Botryosphaeria dieback) are severe diseases affecting grapevines (*Vitis* spp.) worldwide [1,2,3,4,5,6,7]. They dramatically shorten vineyard longevity and compromise their sustainability because the causal pathogens attack long-lasting organs, inducing vine death on the shorter or longer term. From 2003 to 2008, at least 17% of French vineyards showed symptoms of Eutypa dieback [1], with corresponding incidence on wine production and quality. The disease is caused by the ascomycete *Eutypa lata* [8], a random-mating species displaying high genetic diversity [9]. This fungus is also a pathogen for more than 80 other species, including apricot, almond, cherry, apple, olive, peach, pear, ornamental species, and walnut [10,11,12,13]. Symptoms in grapevine include shorter stem internodes, dwarfed and necrotized leaves, some brown and hard sectorial necrosis inside woody parts, and cankers on arms and trunk [13].

The analysis of the diversity and genetic structure of natural populations of *E. lata* [14,15,16] evidenced a large genetic diversity, and a prominent role of ascospores in disease propagation. This diversity for neutral DNA markers has been shown to be accompanied by differences in aggressiveness [4,14,15,17] as well as in metabolite production [18,19]. In particular, differences in aggressiveness among single-spore isolates from the same perithecial stroma led to the identification of pairs of high-aggressive/low-aggressive isolates sharing the same genetic and geographical origins [9]. However, if some research has been conducted on *E. lata* diversity, little information has been published on the susceptibility of grapevines to this pathogen [20,21,22]. The task is very problematic indeed due to the wide variation in symptoms expression and the complex monitoring of fungus growth inside the wood. In order to evaluate the genetic component of variability in grapevine response to *E. lata*, and to other fungi associated with GTD as well, we therefore need rapid and accurate phenotyping tools. In controlled conditions, these tools should provide results in accordance with the behavior of grapevine cultivars in vineyards.

Foliar symptoms induced by one or more toxic metabolites produced by *E. lata* [19,23] are frequently used to detect and monitor infected plants; however, they can drastically vary from one year to another [24,25,26], and are also difficult to reproduce under controlled conditions. Péros and Berger [27] were able to reproduce Eutypa dieback foliar symptoms on plants as early as four weeks after inoculation, and used the percentage of characteristic symptoms and abnormalities appearing at ten weeks to describe the variability in *E. lata* aggressiveness and grapevine cultivars susceptibility. Although these foliar symptoms could be reproduced over the years in this laboratory (Péros, personal communication), other studies using different incubation conditions and isolates repeatedly failed to evidence symptoms, even 11 months after *E. lata* inoculation (e.g., [22]). Therefore, reproducing foliar symptoms on cuttings can hardly be considered an appropriate and repeatable method to evaluate grapevine tolerance to *E. lata*. In parallel, measurements of the necrotic area in the wood of inoculated cuttings have been used by Sosnowski et al. [4] to compare *E. lata* isolates and three grapevine cultivars, and by Travadon et al. [22] to compare susceptibility among eleven cultivars. However, an interval of at least one year was necessary between inoculation and measurements.

Another way to assess Eutypa dieback in inoculated cuttings without measuring external symptoms could be through the use of PCR methods to quantify fungal colonization. Indeed, real-time quantitative (q)PCR has been proposed as a technology enabling the detection and quantification of fungal DNA in plant tissues [28,29]. However, a traditional PCR approach for GTD has mostly been used for the detection or identification of either *E. lata* [30,31] or other fungi such as *Phaemoniella chlamydospora* and *Phaeoacremonium minimum* [32,33,34]. However, Pouzoulet et al. [35] proposed multiplexing different targets to detect and quantify genomic DNA of Esca-associated pathogens in wood samples. This approach could be a good candidate for the precise quantification of *E. lata* mycelium extension after controlled inoculation, and could provide an accurate measurement of variability in both grapevine susceptibility and *E. lata* aggressiveness.

In the present study, our main objective was to develop a method for phenotyping grapevine resistance to *E. lata*, reflecting the actual fungal colonization in the wood. Ideally, the method should be accurate, fungus-specific, and repeatable on a large number of individuals. By comparing high-aggressive and low-aggressive isolates, we also aimed to establish whether there was a link between aggressiveness, foliar symptom expression, and wood colonization, with the perspective of defining the proper strategy for phenotyping Eutypa dieback susceptibility.

## 2. Materials and Methods

### 2.1. Plant Material

Grapevine material from five different grapevine cultivars (cvs. Aramon, Cabernet-Sauvignon, Carignan, Chasselas, and Grenache) was collected in vineyards in winter and stored at +4 °C. According to a previous study, these cultivars display a gradient in the expression of foliar symptoms and abnormalities after inoculation of *E. lata* [27], from cv. Cabernet-Sauvignon (highest percentage of symptomatic plants), to Grenache (lowest percentage), and the other three having intermediate values. Cuttings were prepared according to Péros and Berger [27]. The number of plants is detailed in the Experimental Design section.

### 2.2. Fungal Material and Inoculation

Based on a previous work by Péros and Berger [9], four pairs of high-aggressive and low-aggressive *E. lata* isolates were selected based on their abilities to induce after inoculation foliar symptoms with high or low frequencies, respectively. They were obtained as single ascospores from four different stomata, each pair thus sharing the same genetic and geographical origins (Table 1). Since their collection, isolates were stored in sterile water at +4 °C. For this study, isolates were retrieved from storage and cultured on potato-dextrose agar (PDA) in Petri dishes placed in the dark at 25 °C. After five days, mycelial plugs were taken from the culture margin and inoculated into grapevine cuttings. Inoculations and incubations in the greenhouse were performed according to Péros and Berger [27], and for each experiment controls were included using plugs of PDA medium without fungal culture.

### 2.3. Re-Isolation Method

To determine the extent of *E. lata* mycelium colonization beyond the inoculation point (IP), mycelium was isolated from all plants inoculated with *E. lata*, as well as from the controls. For each treatment, plants were sampled sixteen weeks after bud break. After bark removal, cuttings were surface disinfected as described in Péros and Berger [27]. Thin chips were cut with a scalpel from cross-sections made at different distances from the IP (0, 1, and 2 cm above), and at different times post-inoculation (from 15 to 60 days). Samples were collected at 0 cm (just at the border of the IP) in order to evaluate the inoculation success rate; and at 1 and 2 cm from IP to evaluate plant colonization by *E. lata*. The wood chips were placed onto plates containing a semi-selective PDA medium with 500 mg·L^−1^ chloromycetin and 5 mg·L^−1^ captan [27]. After incubation for 1 week at 25 °C, plates were examined for the presence of *E. lata* mycelium.

### 2.4. Fungal Biomass Measurement by Quantitative PCR

Small pieces of wood were sampled at 1 cm above and below the IP, frozen in liquid nitrogen, and then stored at −80 °C. Wood samples were lyophilized for 48 h and then crushed in a TissueLyser (QIAGEN, Hilden, Germany) using ceramic beads to obtain a fine powder. Genomic DNA was extracted with the Mini-NucleoSpin^R^ Plant II kit (Rev.04, Macherey-Nagel, Düren, Germany) using the PL1 buffer, RNAseA, and 1% PVP (Polyvinylpyrrolidone) for the cell lysis step. DNA quality and concentration were determined with a Nanodrop2000 (Thermo Fisher Scientific, Waltham, Massachusetts, USA) spectrophotometer. Quantitative PCR runs were performed on the StepOnePlus^™^ Real-Time PCR System with Fast Sybr^®^ Green chemistry (Thermo Fisher Scientific Inc.). Primers described in Gatto et al. (2008) [36] were used for amplification of *Vitis vinifera* actin (Table 2). For *E. lata*, we decided to target the β-tubulin gene, because its presence as a single copy in the fungal genome facilitates accurate copy-number measurement by qPCR. Specific primers were designed with the FastPCR software (v.6.3, Primer Digital, Helsinki, Finland) [37] using special settings for quantitative PCR primer design based on β-tubulin sequences available for *E. lata* in NCBI databases (https://www.ncbi.nlm.nih.gov/) (Table 2). Three or four biological replicates (depending on cutting survival), plus three technical replicates were conducted per modality assayed.

PCR reactions were run in a total volume of 20 µL containing: 10 µL Fast SYBR^®^ Green Master Mix, 0.2 µL each primers at 10 µM, 10 ng of DNA, and H_2_O, with the following thermal cycling conditions: 20 s at 95 °C, then 45 cycles of 3 s at 95 °C, and 30 s at 60 °C. The specificity of the individual PCR amplification was checked using a heat dissociation curve from 55 to 95 °C following the PCR final cycle. For absolute quantification of grapevine and *E. lata* genome copy numbers, serial dilutions of external standards (genomic DNA of *Vitis vinifera* cv. Cabernet-Sauvignon and *E. lata* isolate BX1-10, respectively) with known concentrations were used to create standard curves. The standard dilutions were amplified in separate wells, but within the same PCR run. The crossing points of standards and samples were then used to determine the concentration of target DNA. Absolute quantifications were performed with Applied Biosystems StepOnePlus^TM^ software (v2.3, Waltham, Massachusetts, USA). Mean values and standard deviations were obtained from technical and biological replicates.

### 2.5. Experimental Design

Experiment 1: We compared wood colonization by *E. lata* isolates previously characterized for their ability to induce foliar symptoms [9,27] to study the link between foliar symptom expression and wood colonization by mycelium. Comparison of wood colonization by different *E. lata* isolates was assessed by the inoculation of cuttings followed by pathogen re-isolation. Four *E. lata* isolates (AM78-1, AM78-4, VL11-12, and VL11-3) were selected because they presented—within each pair—high contrast in foliar symptoms and abnormalities induction after inoculation [27]. They were separately inoculated to 120 cuttings of cv. Cabernet-Sauvignon (a cultivar known for its susceptibility to Eutypa dieback [27]), and 20 controls were included. Plants were then collected 15, 30, 45, or 60 days after inoculation. On each plant, three wood samples were collected for re-isolation (see below) at 0, 1, and 2 cm from the IP, for a total of 1440 wood samples, corresponding to 30 biological replicates per condition (*E. lata* isolate × dpi × distance from IP).

Experiment 2: Similar experimental conditions were used to compare five different grapevine varieties (cvs. Aramon, Cabernet-Sauvignon, Carignan, Chasselas, and Grenache) for their level of tolerance to wood colonization by the *E. lata* high-aggressive isolate VL11-12. The isolate was inoculated to 80 plants per cultivar, for a total of 400 plants (plus 20 controls per cultivar). A total of 1500 wood samples were collected for re-isolation at 0, 1, and 2 cm from IP, and at 15, 30, 45, or 60 dpi.

Experiment 3: In order to test whether *E. lata* biomass could be monitored, real-time qPCR runs were performed on wood samples collected 1 cm above and below the inoculation point, and at different times after inoculation. Sixteen Cabernet-Sauvignon plants were inoculated with the same isolate VL11-12, and wood samples were collected for DNA extraction from 1 cm above and 1 cm below the IP, at 15, 30, 45, or 60 days after inoculation.

Experiment 4: Finally, we tested the link between the aggressiveness as evaluated by foliar symptoms expression, and the fungal biomass measured in the wood. Three pairs of *E. lata* isolates (AM78-1, AM78-4, BX1-10, BX1-5, CM96-7, and CM96-6) were separately inoculated to four cuttings of cv. Cabernet-Sauvignon, for a total of 24 plants. Wood samples were collected on each cutting at 1 cm above the IP at 10 weeks after inoculation to apply the real-time PCR procedure.

### 2.6. Data Analysis and Reproducible Research

Data were analyzed and visualized within the R software environment [38] using RStudio [39], and R Markdown formatted files combining the core syntax of markdown with embedded R code chunks (see http://rmarkdown.rstudio.com/index.html for more details). Raw data and R Markdown files are provided in supplementary data, allowing anyone to reproduce the analysis and figures presented in this manuscript [40]. Tests for equality of proportions (experiments 1 and 2) were performed using the Bayesian-First-Aid package [41]. For analysis of variance (experiments 3 and 4), the models were first fitted with *aov* function from R package *stats* (v.3.2.4), and model validity was assessed by inspecting the residuals (for details, see Rmd file in supplementary data).

## 3. Results

### 3.1. Wood Colonization in Relation to Aggressiveness and Distance from Inoculation Site (Experiment 1)

None of the wood samples from control plants was found colonized by *E. lata*. In plants inoculated with *E. lata*, 28% of wood samples were contaminated with fungi other than *E. lata* (Figure S1). Such contaminations were equally distributed among the different treatments, except for samples treated with isolate VL11-3, which resulted in more contaminations. *E. lata* can be concealed by other fungi present in the wood chips and growing faster on PDA medium, preventing its development and identification. Therefore, all contaminated samples were removed from the following analysis. In the case of isolate VL11-3, its low-aggressiveness might entail a weaker development that allowed the development of other fungi, amplifying the number of contaminated samples. The rest of the samples were either considered as “uncontaminated” when no *E. lata* was observed or “colonized by *E. lata*” if the fungus was identified after sub-culture on PDA medium.

**Figure 1:**
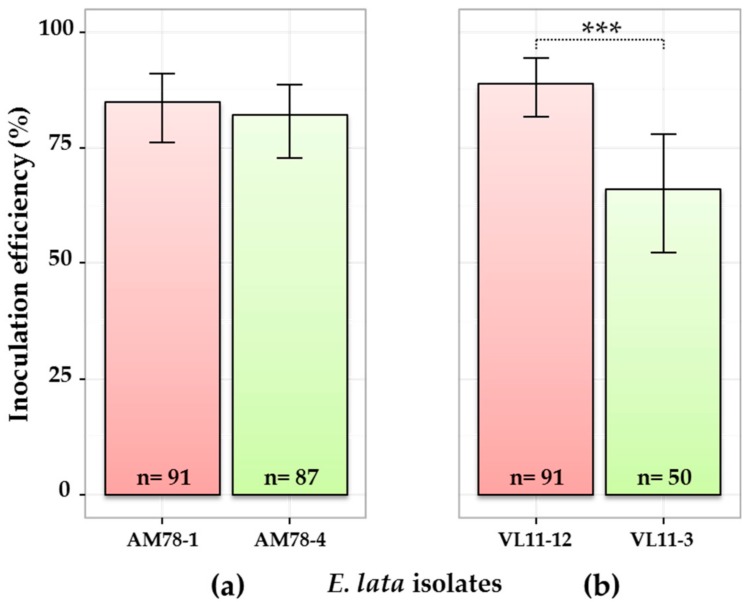
Comparison of four *E. lata* isolates for inoculation efficiency. Pair of isolates from (**a**) stroma AM78 and (**b**) stroma VL11 inoculated to cv. Cabernet-Sauvignon. Here, only samples collected at the border (0 cm) of the inoculation point are considered (other contaminations excluded). High- and low-aggressive (according to Péros and Berger [27]) isolates are shown in red and green, respectively. *n*: number of observations; 95% credible intervals were calculated for each isolate using a Bayesian proportion test and represented as error bars in the figure. Asterisks *** indicate that the relative frequency of success is significantly different between isolates, with a >0.999 probability.

As expected, wood samples collected at 1 cm above the IP were more frequently colonized by *E. lata* than those collected at 2 cm (Figure S1). Generally, inoculations performed with high-aggressive isolates were more efficient in term of percentage of infected plants than inoculations performed with low-aggressive isolates (Figure 1). However, the contrast between VL11-12 (89% inoculation success) and VL11-3 (66%) (Figure 1b) was higher than between AM78-1 (85%) and AM78-4 (82%) (Figure 1a). Testing for equality of proportions in a pairwise manner between isolates originated from the same stroma allowed us to conclude that the estimated relative frequency of inoculation success was significantly different, with a 0.999 probability between the two VL isolates, but not between the two AM isolates.

**Figure 2:**
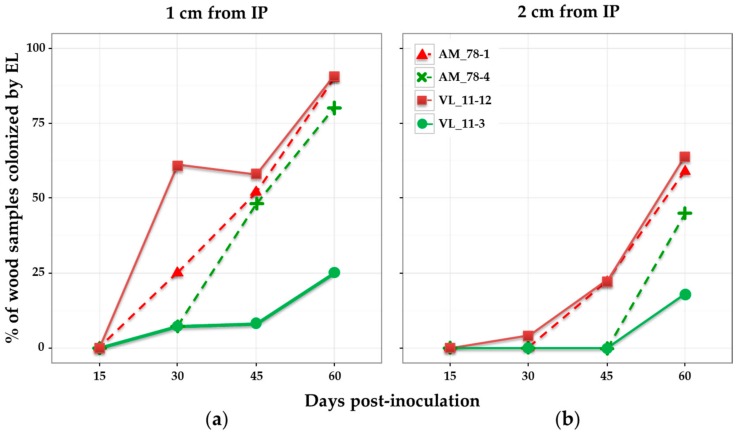
Percentage of wood samples colonized by four *E. lata* isolates. Re-isolation from wood samples collected on cv. Cabernet-Sauvignon at: (**a**) 1 cm; and (**b**) 2 cm from the inoculation point; and at different days post-inoculation. High- and low-aggressive (according to Péros and Berger [27]) isolates are shown in red and green, respectively.

When considering the percentage of wood samples colonized after different times post-inoculation, results showed different patterns depending on the isolate (Figure 2). None of the samples was colonized by *E. lata* after 15 days. This lapse of time seemed too short for the fungus to be present 1 cm above the IP. After 30 days post-inoculation, high-aggressive isolates AM78-1 and VL11-12 were more often detected in wood samples than their low-aggressive relatives AM78-4 and VL11-3, respectively (Figure 2). The high-aggressive isolates AM78-1 and VL11-12 showed similar patterns for wood colonization. They were detected in more than 25% of the samples collected at 1 cm after 15 days, and in 22% of those collected at 2 cm after 30 days. Their progression in wood was regular, reaching 90% of samples colonized at 1 cm, and approx. 60% at 2 cm after 60 days. The low-aggressive isolate AM78-4 was detected in 8% of the samples collected at 1 cm after 30 days, in 48% after 45 days and 80% after 60 days. It was not present at 2 cm at 45 days, but eventually detected in 45% of the samples after 60 days at this distance. By contrast, the low-aggressive isolate VL11-3 was rarely detected, with a maximum of 25% of the samples colonized at 1 cm and 18% at 2 cm after 60 days.

### 3.2. Comparison of Grapevine Cultivars for Their Tolerance to Wood Colonization by E. lata (Experiment 2)

In the second experiment, five grapevine varieties were compared for their tolerance to the spread of an aggressive isolate in their wood. Among the 300 wood samples collected on control plants of the five cultivars tested, 41.7% were contaminated with other fungi and distributed over the days post-inoculation (Figure S2), while 58.3% were uncontaminated. Among the 1200 samples of wood collected on inoculated plants, 22% were contaminated with fungi other than *E. lata* (Figure S3). External contaminations equally affected samples collected at different distances from IP, except for cv. Carignan, where samples collected at the border of IP (0 cm) were contaminated twice as often at 1 and 2 cm.

**Figure 3:**
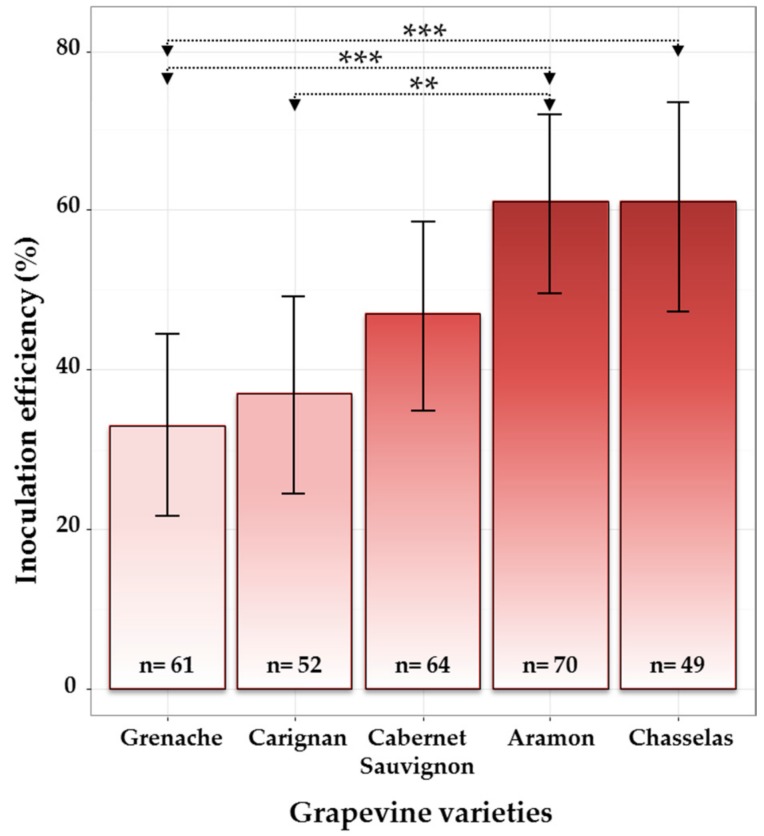
Effect of grapevine cultivar on the infection success in cuttings inoculated with *E. lata,* isolate VL11-12. Considering samples collected at the border (0 cm) of the inoculation point (contaminated samples excluded, n: number of observations); 95% credible intervals were calculated for each cultivar using Bayesian proportion test and are represented as error bars in the figure. Asterisks *** and ** indicate that the relative frequencies of success are significantly different between cultivars with a probability of >0.999 and >0.997, respectively.

As expected, wood samples collected at 1 cm from the IP were more frequently colonized by *E. lata* than those collected at 2 cm (Figure S3). All contaminated samples were removed from the analysis before estimating wood colonization by *E. lata* and calculating inoculation efficiencies. Considering the presence of *E. lata* at 0 cm from the IP, the five cultivars showed different degrees of tolerance to infection with *E. lata* isolate VL11-12. Indeed, percentages of inoculation success varied from 33% for cv. Grenache to 61% for both cv. Aramon and cv. Chasselas, with cv. Carignan and cv. Cabernet-Sauvignon showing intermediate values (Figure 3). Testing for equality of proportions between all five cultivars allowed us to conclude that there was a significant effect of grapevine cultivar (at the 5% level) on the infection success (*p*-value = 0.002). When testing in a pairwise manner, cv. Aramon was significantly more infected than both cvs. Grenache (posterior probability, *PP* = 0.999) and Carignan (*PP* = 0.997), whereas cv. Chasselas was significantly more infected than cv. Grenache (*PP* = 0.998) (see distributions of posterior probabilities in supplementary data).

**Figure 4:**
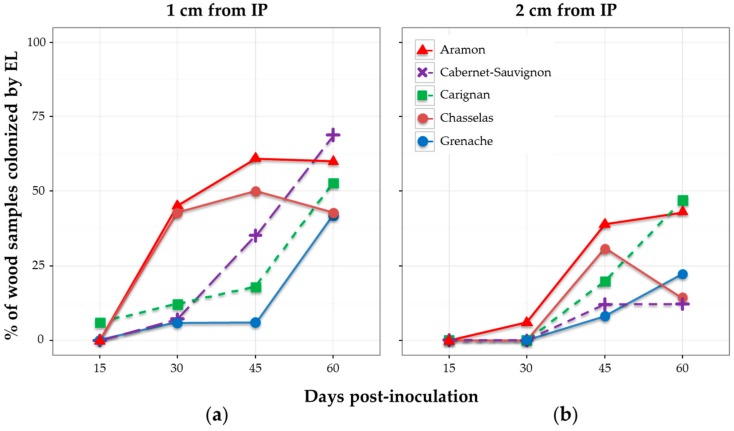
Effect of grapevine cultivar on the wood colonization in plants inoculated with *E. lata* (EL) isolate VL11-12. Wood samples were collected at: (**a**) 1 cm; and (**b**) 2 cm from the inoculation point; and at different days post-inoculation.

When considering the percentage of wood samples colonized after 15, 30, 45, and 60 days, results showed different patterns depending on the cultivar (Figure 4). None of the samples was colonized by *E. lata* after 15 days, except for one single cutting of cv. Carignan. Considering the distance of 1 cm from IP, cultivars cv. Aramon and cv. Chasselas showed similar patterns of colonization by *E. lata*—approx. 45% of plants infected at 30 dpi, and a maximum (61% and 50%) reached at 45 dpi (Figure 4). The diminution in the percentage of infected cv. Chasselas samples observed at 60 dpi might be a consequence of the higher number of external contaminations at this date (Figure S3). Aramon and Cabernet-Sauvignon were the cultivars showing the highest percentages of wood infected by *E. lata* at 60 dpi (60% and 69%, respectively). By contrast, *E. lata* was rarely detected in cv. Carignan and cv. Grenache after 45 dpi. At 60 dpi, approximately the same percentages of infected wood were observed at 1 cm in all five cultivars tested.

Observations made at 2 cm gave similar profiles, with a 15-day delay in comparison with the 1-cm distance for cv. Aramon and cv. Chasselas, and lower percentages of colonization by *E. lata* for cv. Cabernet-Sauvignon and cv. Grenache (Figure 4).

### 3.3. qPCR as a Tool to Evaluate Wood Colonization by E. lata (Experiment 3)

In the third experiment, wood colonization by *E. lata* of cv. Cabernet-Sauvignon plants was monitored by fungal biomass measurement using qPCR and fungus- and plant-specific primers (Table 2). Performing qPCR using β-tubulin primers on wood DNA or using *V. vinifera* actin primers on DNA extracted from *E. lata* mycelium did not result in any signal (data not shown), proving the specificity of both PCR amplifications.

**Figure 5:**
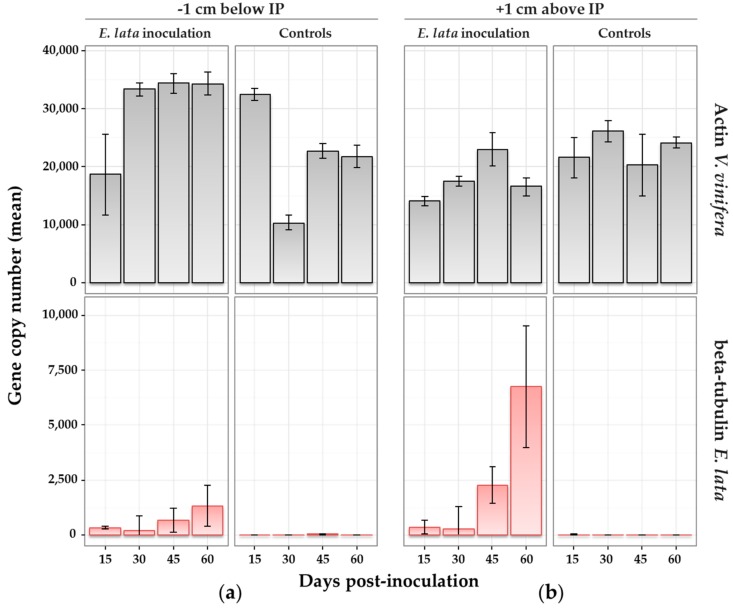
Wood colonization by *E. lata* monitored by quantitative real-time PCR (qRT-PCR). Samples collected on cv. Cabernet-Sauvignon at (**a**) 1 cm above and (**b**) 1 cm below the inoculation point (IP) at different number of days after inoculation. Treatment (inoculation with *E. lata*, or with sterile peptone-dextrose agar (PDA) medium for controls) is indicated in the top caption. qPCR target (actin from *V. vinifera* in grey or β-tubulin from *E. lata* in red) is indicated in the right-side caption. Standard error bars indicate range of values for biological and technical replicates.

Differences between biological replicates for the quantity of actin amplicons were observed, indicating variability in grapevine gDNA extraction from wood samples (Figure 5). However, this did not alter the results for *E. lata*, since the quantity of actin was not correlated to the number of β-tubulin quantified, and therefore to the corresponding fungal biomass measurement. Using β-tubulin primers, PCR performed on DNA samples extracted from control plants (above and below the IP) gave no detectable signal, confirming the absence of the pathogen (Figure 5).

Within plants inoculated with *E. lata*, β-tubulin was amplified after 15 days post-inoculation. In samples collected above and below the IP (Figure 5), β-tubulin amplicons were generally at low copy numbers at 15 and 30 days post-inoculation, and within similar ranges (means from 185 to 349 copies). At 45 days, the quantity of *E. lata* was larger in samples collected 1 cm above (mean over 2000 copies) than below (mean at 667) the IP. This difference was even clearer at 60 days, with a mean of 6750 copies detected above the IP, versus 1330 below (Figure 5). Results were similar when considering the ratio β-tubulin/actin (Figure S4), indicating that DNA extraction efficiency did not influence results. The analysis of variance suggested that both the distance from IP and time post-inoculation had a significant effect at the 5% level on the detected quantity of *E. lata* (*p*-values = 0.0233 and 0.0038, respectively). In other words, the quantity of *E. lata* measured above and below the IP significantly increased over time. The interaction of time and distance effects was also significant at the 5% level (*p*-value = 0.0181), meaning that the quantity of fungus measured at each distance from the IP was increasing over time at different speeds.

### 3.4. Comparison of E. lata Aggressiveness by qPCR (Experiment 4)

**Figure 6:**
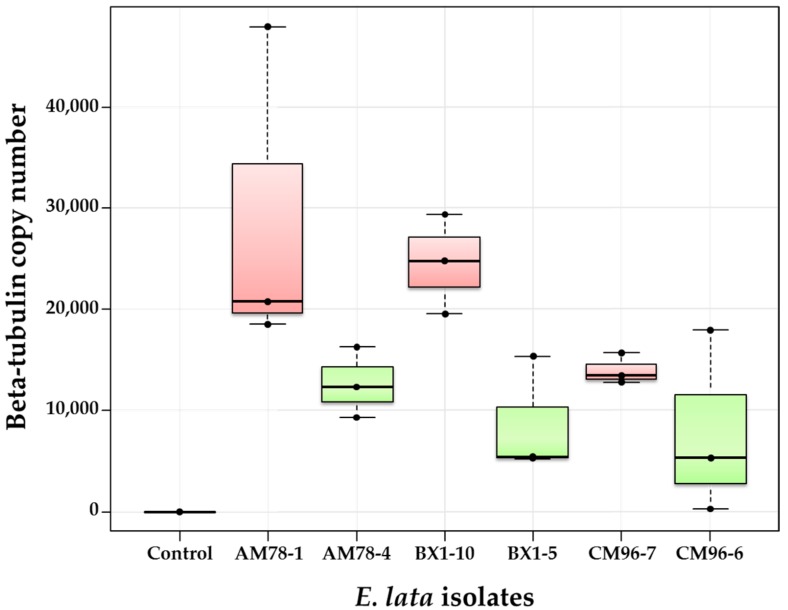
Comparison of wood colonization by high-aggressive and low-aggressive isolates of *E. lata*, using biomass measurements through real-time PCR. Wood samples were collected ten weeks after inoculation at 1 cm above the IP on cv. Cabernet-Sauvignon. High- and low-aggressive (according to Péros and Berger [27]) isolates are shown in red and green, respectively.

In this fourth experiment, inoculated plants of cv. Cabernet-Sauvignon were grown for ten weeks after inoculation to allow better wood colonization of *E. lata*, in order to increase differences between phenotypes and to facilitate quantification by qPCR. The number of beta-tubulin copies measured at 1 cm from the IP for BX1-10 isolate was indeed two-to-three times higher 10 weeks post-inoculation (Figure 6) than it was after 60 days in the previous experiment (Figure 5). As expected, β-tubulin was not amplified from control samples, although it was amplified from all 18 plants inoculated with *E. lata* (Figure 6). Median copy numbers of *E. lata* β-tubulin measured in plants inoculated with high-aggressive isolates AM78-1, BX1-10, and CM96-7 were all higher than those measured with their low-aggressive relatives AM78-4, BX1-5, and CM96-6 (Figure 6). Results also revealed that isolates AM78-1 and BX1-10 had higher wood colonization efficacy than the high-aggressive isolate CM96-7. Inoculations with the isolate CM96-6 resulted in contrasting results, beta-tubulin copy numbers ranging from 273 to 17,935 among plants. The analysis of variance indicated that wood colonization was significantly linked to aggressiveness at the 5% level (*p*-value = 0.007). Plants inoculated with high-aggressive isolates were always more heavily infected than those inoculated with low-aggressive isolates, confirming the link between wood colonization and foliar symptom expression as previously described [9]. However, there was neither a significant effect of stroma origin (*p*-value = 0.163), nor a significant interaction between stroma and aggressiveness as pre-evaluated by foliar symptoms expression (*p*-value = 0.508).

## 4. Discussion

The observation of GTD external symptoms is one way to monitor trunk disease and assess cultivar susceptibility, but they are difficult to reproduce under controlled conditions and are extremely irregular in their expression [24,25,26,42]. We sought an approach that could be applied in genome-wide association studies involving a large genetic diversity, allowing both the measure of fungal wood colonization and the phenotyping of numerous grapevine varieties for their tolerance. The measurement of necrotic areas in wood has technical limitation, since it often requires several months to ensure proper monitoring. For *Eutypa* dieback, the extent of wood discoloration was measured 11 months after inoculation in order to compare susceptibility in 11 grapevine cultivars [22]. Another study measured the extent of staining induced by *E. lata*, and performed re-isolations 20–24 months after inoculation in order to compare different isolates and cultivars [4]. Another limitation is that the necrotic area might reflect the wood colonizing capability of a fungus through its production of metabolites and toxins, but might incorrectly estimate both its colonization rate and the damages caused to the wood. Indeed, Sosnowski et al. [4] observed that the staining of wood tissues attributed to *E. lata* in their experiments did not correspond to its actual presence, because the fungus was detected up to 8 cm beyond the stain. Moreover, the severity of foliar symptoms induced by *E. lata* infection was not related to the rate of spread of the fungus when estimated by necrotic area observations [4].

We tested two methods to phenotype resistance of grapevines to *E. lata*, reflecting actual fungal colonization in the wood of infected plants, and aimed at establishing whether there is a link between aggressiveness and wood colonization.

The variation in pathogenicity between several *E. lata* isolates has already been studied based on their ability to induce foliar symptoms [9,27]. In the present study, some previously characterized isolates have been used to evaluate the relation between wood colonization and foliar symptoms induction. Colonization patterns deduced from re-isolation were similar for both high-aggressive isolates of *E. lata*, with a 15-days delay between 1 cm and 2 cm. The low-aggressive isolate AM78-4 showed a short delay in wood colonization compared with the high-aggressive isolates, whereas another low-aggressive isolate VL11-3 failed in colonizing most of the plants. There was a clear difference between VL11-12 (an isolate that induced foliar symptoms at high frequency and efficiently colonized the wood) and VL11-3, an isolate that rarely induced foliar symptoms and slowly expanded in the wood. By contrast, the distinction between AM isolates for their ability to colonize wood was not as obvious when using a re-isolation approach. For this purpose, qPCR might be the appropriate tool to highlight differences that cannot be evidenced by other approaches.

The tolerance of grapevine cultivars to *E. lata* colonization was also assessed by a re-isolation approach. We observed an effect of grapevine cultivar on the infection success in plants inoculated with *E. lata*. It has been proposed that differences in cultivar susceptibility to pruning-wound infections by *E. lata* were highly correlated to the rates of suberin and lignin deposition after wood injuries [43], which might explain differences in inoculation success in our experiment. Re-isolation of *E. lata* from wood samples collected at different times and distances from the IP did not point to any clear differences between cultivars, with all five cultivars exhibiting similar patterns of fungus propagation. *E. lata* might have spread more rapidly in cv. Aramon and Chasselas than in other cultivars, colonizing almost one half of the plants at 1 cm after 45 days, while cv. Cabernet-Sauvignon, Carignan, and Grenache only reached similar values after 60 days. However, it was otherwise difficult to extract clear information on the degree of tolerance of these cultivars using a re-isolation approach. For example, the high susceptibility of cv. Chasselas was confirmed, but the rapid colonization of cv. Aramon by *E. lata* did not correlate to the low level of symptoms observed in a previous study in the same controlled conditions [27]. The number of contaminations with other fungi might have affected the amount of data available, and therefore their interpretation. Finally, the re-isolation of *E. lata* from wood samples collected on inoculated plants did not provide any quantitative values that could be mined to detect and evaluate the potential genetic origin of tolerance or resistance traits.

Real-time PCR might be more appropriate to highlight differences that could go unseen using re-isolation. In order to test whether wood colonization by *E. lata* could be monitored through fungal biomass quantification, measurements of *E. lata* biomasses were performed by qPCR on wood samples collected above and below the IP, and at different times after inoculation. Using β-tubulin—a single-copy gene per haploid genome—as target sequence for PCR amplification provided a more reliable quantification of fungal DNA [29]. Detection and quantification by qPCR had already been tested for two fungi (*P. chlamydospora* and *P. minimum*) associated with GTD [35], but not for the Eutypa dieback agent. *E. lata* is able to produce enzymes attacking cell walls, degrading lignin and starch in parenchymatous rays [44,45]. Certain effectors of pathogenic fungi (e.g., toxins) can stimulate the destruction and relocation of actin filaments [46], and could therefore have disturbed the detection of the *V. vinifera* actin gene. However, the number of actin copies detected by qPCR in our experiments did not seem to be influenced by the presence of *E. lata* in collected samples. The degradation of grapevine tissues during *E. lata* colonization did not alter the detection or quantification of fungal biomass.

Variability in *E. lata* β-tubulin copy numbers was more important between biological replicates than the variability in *V. vinifera* actin copy number, indicating heterogeneity in *E. lata* colonization between plants. Several explanations can be proposed, such as the diversity of *E. lata* itself, naturally varying between isolates in its ability to progress through the wood [4], but also differences in wood structure from one cutting to another, in term of cell development, tissues, and vessels. It has already been suggested that vessel dimension could explain tolerance toward fungal vascular wilt diseases in woody plants, based on the observation that the susceptibility of three *V. vinifera* cultivars to esca disease was correlated to large vessel diameter [47]. Based on this assumption, even tiny between-plant variations in the architecture of vessels or cells could affect fungal colonization, and therefore explain biomass measurement heterogeneity among plants. Plant culture for ten weeks after inoculation appeared to be a proper timing for collecting samples when comparing *E. lata* isolates for aggressiveness. The development of *E. lata* in the wood was long enough to reach a distance of 1 cm from the IP, and it had produced a sufficient quantity of mycelium to be measured by qPCR and to highlight differences between *E. lata* isolates strongly differing in aggressiveness.

Finally, fungal biomass quantification through qPCR allowed the monitoring of *E. lata* progress in the wood and permitted the discrimination between *E. lata* isolates for their aggressiveness when other approaches failed to highlight them. High-aggressive isolates were always more abundant at 1 cm, and therefore more efficient in wood colonization than their related low-aggressive isolates, although they both shared the same genetic and geographical origins [9]. On one hand, qPCR cost time for samples preparation (collection, gDNA extraction, qPCR measurement) and was more expensive than a simple re-isolation of fungus on PDA plates. On the other hand, qPCR was an accurate tool, as it provided a quantitative estimate of fungal biomass using a limited number of samples compared to re-isolation. Fungal biomass measurement by qPCR does not seem appropriate for pathogen detection, since re-isolation or traditional PCR are sufficient for that purpose. However, the qPCR tool proved useful for phenotyping fungal aggressiveness in wood colonization and grapevine tolerance. It could easily be adapted to other fungi involved in GTD by designing PCR primers and amplification conditions adapted to each fungus. A further step would be to use such tools to evaluate the genetic part of tolerance to Eutypa dieback and other grapevine trunk diseases by association genetics using large diversity panels of cultivars.

## 5. Conclusions

These experiments proved that re-isolation is an appropriate tool to monitor wood colonization by *E. lata*, providing consistent results more rapidly than foliar symptoms observation or necrotic areas measurements. However, re-isolation was not quantitative, and was affected by other microorganisms that possibly concealed the presence of *E. lata*.

The qPCR approach showed for the first time a correlation between *E. lata*‘s abilities to induce foliar symptoms and to colonize wood. This correlation has been confirmed by both re-isolation and biomass measurement through qPCR. Indeed, aggressive isolates of *E. lata* which induced more foliar symptoms have also shown higher capabilities to colonize wood than isolates leading to low symptom expression. More specifically, genomic copy number quantification by qPCR enabled the discrimination between AM isolates while the re-isolation approach was not effective.

The qPCR approach may prove to be a useful technology to evaluate grapevine susceptibility to *E. lata* invasion and could be adapted to other pathogens associated with grapevine trunk diseases [35]. In vineyards, grapevine susceptibility to Eutypa dieback may result from several external factors, such as weather conditions, plant reserves, or biotic and abiotic stress. However, wood colonization by *E. lata* is probably a very important step that could be assessed in conditions where other factors are under control. This approach will be used later to evaluate a core collection of *V. vinifera* cultivars for their tolerance to fungal colonization. An association-genetics approach will then be performed in order to identify regions in the grapevine genome associated with variation in tolerance. This could possibly lead to a better knowledge of plant–pathogen interactions and to the development of new genetic markers to improve and speed-up breeding and selection.

## Figures and Tables

**Table 1:** Origin and level of aggressiveness of the *E. lata* isolates used in this study, from Péros and Berger [27].

Stroma	Location	Year of Isolation	High-Aggressive Isolate	Low-Aggressive Isolate
AM78	Aigues-Mortes	1992	AM78-4	AM78-1
BX1	Bordeaux	1990	BX1-10	BX1-5
CM96	Chambolle-Musigny	1993	CM96-7	CM96-6
VL11	Villeneuve-lès-Maguelone	1992	VL11-12	VL11-3

**Table 2:** Quantitative PCR primers used to assess grapevine wood colonization by *E. lata*.

Primers	Sequences (5’ -> 3’)		Length (nt)	Target	PCR Product Size	Organism	Source
Act3_37_F	TCCTTGCCTTGCGTCATCTAT	Fwd	21	Actin	72 bp	*V. vinifera*	Gatto et al. [36]
Act3_38_R	CACCAATCACTCTCCTGCTACAA	Rev	23				
EL_b-tub_33_F	CTGGCAATGCTAACTCGCCGCT	Fwd	22	β-tubulin	90 bp	*E. lata*	This study
EL_b-tub_34_R	CGAGGAACATACTTGTTGCCGGAC	Rev	24				

## Supplementary Materials

The following are available online at www.mdpi.com/2309-608X/3/2/21/s1. Figure S1: Comparison of four isolates of *E. lata* for their ability to colonize grapevine wood; Figure S2: Contaminations observed in wood chips sampled in control plants; Figure S3: Comparison of grapevine cultivars for their tolerance to wood colonization by *E. lata* and other microorganisms; Figure S4: Wood colonization by *E. lata* monitored by qRT-PCR: ratio β-tubulin/actin. Supplementary file “Analysis_and_Figures_for_Manuscript.zip”: containing raw data (data_AGR.csv, EL_wood_compare_4_isolates.txt, EL_wood_compare_5_cultivars_Vv.txt, and EL_qPCR_different_points.txt), and the R Markdown file “Analysis_and_Figures_for_Manuscript.Rmd” (plus its conversion to html and pdf), allowing anyone to reproduce the analysis and figures.

## References

[1] Bruez E., Lecomte P., Grosman J., Doublet B., Bertsch C., Fontaine F., Ugaglia A., Teissedre P.L., Da Costa J.P., Guerin-Dubrana L. (2013). Overview of grapevine trunk diseases in France in the 2000s. Phytopathol. Mediterr..

[2] Mugnai L., Graniti A., Surico G. (1999). Esca (Black Measles) and Brown Wood-Streaking: Two Old and Elusive Diseases of Grapevines. Plant Dis..

[3] Reisenzein H., Berger N., Nieder G. (2000). Esca in Austria. Phytopathol. Mediterr..

[4] Sosnowski M.R., Lardner R., Wicks T.J., Scott E.S. (2007). The influence of grapevine cultivar and isolate of Eutypa lata on wood and foliar symptoms. Plant Dis..

[5] Úrbez-Torres J.R., Leavitt G.M., Voegel T.M., Gubler W.D. (2006). Identification and distribution of *Botryosphaeria* spp. associated with grapevine cankers in California. Plant Dis..

[6] White C.L., Halleen F., Mostert L. (2011). Symptoms and fungi associated with esca in South African vineyards. Phytopathol. Mediterr..

[7] Bertsch C., Ramírez-Suero M., Magnin-Robert M., Larignon P., Chong J., Abou-Mansour E., Spagnolo A., Clément C., Fontaine F. (2013). Grapevine trunk diseases: Complex and still poorly understood. Plant Pathol..

[8] Rappaz F. (1984). Sanctioned species of the genus Eutypa (Diatrypaceae, ascomycetes)—Taxonomic and nomenclatural study. Mycotaxon.

[9] Péros J., Berger G. (1999). Diversity within natural progenies of the grapevine dieback fungus *Eutypa lata*. Curr. Genet..

[10] Carter M.V., Bolay A., Rappaz F. (1983). An annotated host list and biblography of *Eutypa armeniacae*. Rev. Plant Pathol..

[11] Glawe D.A., Rogers J.D. (1982). Observations on the anamorphs of six species of *Eutypa and Eutypella*. Mycotaxon.

[12] Trouillas F.P., Gubler W.D. (2010). Host range, biological variation, and phylogenetic diversity of *Eutypa lata* in California. Phytopathology.

[13] Carter M.V. (1991). The Status of Eutypa Lata as a Pathogen.

[14] Péros J.P., Berger G., Lahogue F. (1997). Variation in Pathogenicity and Genetic Structure in the Eutypa lata Population of a Single Vineyard. Phytopathology.

[15] Péros J.P., Berger G. (2003). Genetic Structure and Variation in Aggressiveness in European and Australian Populations of the Grapevine Dieback Fungus, Eutypa lata. Eur. J. Plant Pathol..

[16] Travadon R., Baumgartner K., Rolshausen P.E., Gubler W.D., Sosnowski M.R., Lecomte P., Halleen F., Péros J.P. (2012). Genetic structure of the fungal grapevine pathogen *Eutypa lata* from four continents. Plant Pathol..

[17] DeScenzo R.A., Engel S.R., Gomez G., Jackson E.L., Munkvold G.P., Weller J., Irelan N.A. (1999). Genetic analysis of *Eutypa* strains from california supports the presence of two pathogenic species. Phytopathology.

[18] Molyneux R.J., Mahoney N., Bayman P., Wong R.Y., Meyer K., Irelan N. (2002). *Eutypa* Dieback in Grapevines: Differential production of acetylenic phenol metabolites by strains of *Eutypa lata*. J. Agric. Food Chem..

[19] Mahoney N., Lardner R., Molyneux R.J., Scott E.S., Smith L.R., Schoch T.K. (2003). Phenolic and heterocyclic metabolite profiles of the grapevine pathogen Eutypa lata. Phytochemistry.

[20] Dubos B. (1987). Mise au point sur les maladies de dépérissement dans le vignoble francais. Compte-rendu de la réunion du groupe de travail sur les maladies de dépérissement de la vigne eutypiose (*Eutypa Armenicae*), esca (*Stereum hirsutum*, *Phellinus* sp.). Progrès Agric. Vitic..

[21] Chapuis L., Richard L., Dubos B. (1998). Variation in susceptibility of grapevine pruning wound to infection by Eutypa lata in south-western France. Plant Pathol..

[22] Travadon R., Rolshausen P.E., Gubler W.D., Cadle-Davidson L., Baumgartner K. (2013). Susceptibility of cultivated and wild *Vitis* spp. to wood infection by fungal trunk pathogens. Plant Dis..

[23] Octave S., Roblin G., Vachaud M., Fleurat-Lessard P. (2006). Polypeptide metabolites secreted by the fungal pathogen *Eutypa lata* participate in *Vitis vinifera* cell structure damage observed in Eutypa dieback. Funct. Plant Biol..

[24] Sosnowski M.R., Shtienberg D., Creaser M.L., Wicks T.J., Lardner R., Scott E.S. (2007). The influence of climate on foliar symptoms of eutypa dieback in grapevines. Phytopathology.

[25] Dumot V., Ménard E., Courlit Y., Ouvrie M., Desache F., Boursier N., David S., Dubos B., Larignon P. (2004). *Eutypa canker* in the Charentes region (France). Results of a 10-year study on *Ugni blanc*. Phytoma.

[26] Creaser M., Wicks T. (2001). Yearly variation in Eutypa dieback symptoms and the relationship to grapevine yield. Aust. Grapegrow. Winemak..

[27] Péros J.-P., Berger G. (1994). A rapid method to assess the aggressiveness of *Eutypa lata* isolates and the susceptibility of grapevine cultivars to Eutypa dieback. Agronomie.

[28] Schena L., Nigro F., Ippolito A., Gallitelli D. (2004). Real-time quantitative PCR: A new technology to detect and study phytopathogenic and antagonistic fungi. Eur. J. Plant Pathol..

[29] Atallah Z.K., Bae J., Jansky S.H., Rouse D.I., Stevenson W.R. (2007). Multiplex real-time quantitative PCR to detect and quantify *Verticillium dahliae* colonization in potato lines that differ in response to verticillium wilt. Phytopathology.

[30] Lecomte P., Péros J.P., Blancard D., Bastien N., Delye C. (2000). PCR assays that identify the grapevine dieback fungus Eutypa lata. Appl. Environ. Microbiol..

[31] Camps C., Kappel C., Lecomte P., Leon C., Gomes E., Coutos-Thevenot P., Delrot S. (2010). A transcriptomic study of grapevine (*Vitis vinifera* cv. Cabernet-Sauvignon) interaction with the vascular ascomycete fungus Eutypa lata. J. Exp. Bot..

[32] Edwards J., Constable F., Wiechel T., Salib S. (2007). Comparison of the molecular tests-single PCR, nested PCR and quantitative PCR (SYBR^®^ Green and TaqMan^®^)-for detection of Phaeomoniella chlamydospora during grapevine nursery propagation. Phytopathol. Mediterr..

[33] Overton B.E., Stewart E.L., Qu X., Wenner N.G., Christ B.J. (2004). Qualitative real-time PCR SYBR Green detection of Petri disease fungi. Phytopathol. Mediterr..

[34] Martin M.T., Cobos R., Martin L., Lopez-Enriquez L. (2012). Real-Time PCR Detection of Phaeomoniella chlamydospora and Phaeoacremonium aleophilum. Appl. Environ. Microbiol..

[35] Pouzoulet J., Mailhac N., Couderc C., Besson X., Daydé J., Lummerzheim M., Jacques A. (2013). A method to detect and quantify Phaeomoniella chlamydospora and Phaeoacremonium aleophilum DNA in grapevine-wood samples. Appl. Microbiol. Biotechnol..

[36] Gatto P., Vrhovsek U., Muth J., Segala C., Romualdi C., Fontana P., Pruefer D., Stefanini M., Moser C., Mattivi F. (2008). Ripening and genotype control stilbene accumulation in healthy grapes. J. Agric. Food Chem..

[37] Kalendar R., Lee D., Schulman A.H. (2009). FastPCR software for PCR primer and probe design and repeat search. Genes Genomes Genom..

[38] R Core Team (2016). R: A Language and Environment for Statistical Computing.

[39] RStudio Team (2016). RStudio: Integrated Development for R.

[40] Goodman S.N., Fanelli D., Ioannidis J.P.A. (2016). What does research reproducibility mean?. Sci. Transl. Med..

[41] Bååth R. Bayesian First Aid: A Package that Implements Bayesian Alternatives to the Classical * .test Functions in R. Proceedings of the UseR! 2014-the International R User Conference.

[42] Surico G., Marchi G., Braccini P., Mugnai L. (2000). Epidemiology of esca in some vineyards in Tuscany (Italy). Phytopathol. Mediterr..

[43] Munkvold G., Marois J. (1995). Factors associated with variation in susceptibility of grapevine pruning wounds to infection by *Eutypa lata*. Phytopathol. Mediterr..

[44] del Río J.A., Gómez P., Báidez A., Fuster M.D., Ortuño A., Frías V. (2004). Phenolic compounds have a role in the defence mechanism protecting grapevine against the fungi involved in Petri disease. Phytopathol. Mediterr..

[45] Rudelle J., Octave S., Kaid-Harche M., Roblin G., Fleurat-Lessard P. (2005). Structural modifications induced by *Eutypa lata* in the xylem of trunk and canes of *Vitis vinifera*. Funct. Plant Biol..

[46] Yuan H.Y., Yao L.L., Jia Z.Q., Li Y., Li Y.Z. (2006). Verticillium dahliae toxin induced alterations of cytoskeletons and nucleoli in *Arabidopsis thaliana* suspension cells. Protoplasma.

[47] Pouzoulet J., Pivovaroff A.L., Santiago L.S., Rolshausen P.E. (2014). Can vessel dimension explain tolerance toward fungal vascular wilt diseases in woody plants? Lessons from Dutch elm disease and esca disease in grapevine. Front. Plant Sci..

