# Supplementary material for: Quantitative Assessment of Grapevine Wood Colonization by the Dieback Fungus Eutypa lata
Source: J Fungi (Basel). 2017 May 6;3(2):21. doi: 10.3390/jof3020021 (PMC5715921; doi:10.3390/jof3020021)
Supplement: Supplementary file 1 [file jof-03-00021-s001.zip › Moisy_et_al__Supplementary_figures.docx]

Type of the Paper : Article

« Quantitative assessment of grapevine wood colonization by the dieback fungus *Eutypa lata* »

Cédric Moisy ^1,^*, Gilles Berger ^2^, Timothée Flutre ^2^, Loïc Le Cunff ^1^, and Jean-Pierre Péros ^2^

^1^ Institut Français de la Vigne et du Vin, UMT Géno-Vigne, F-34060 Montpellier, France ; cedric.moisy@laposte.net

^2^ INRA, UMR AGAP, F-34060 Montpellier, France; jean-pierre.peros@inra.fr

***** Correspondence: cedric.moisy@laposte.net; Tel.: +33-(0)499612774

Academic Editor: name

Received: date; Accepted: date; Published: date

**Supplementary Materials:** The following are available online at [www.mdpi.com/link](http://www.mdpi.com/link)


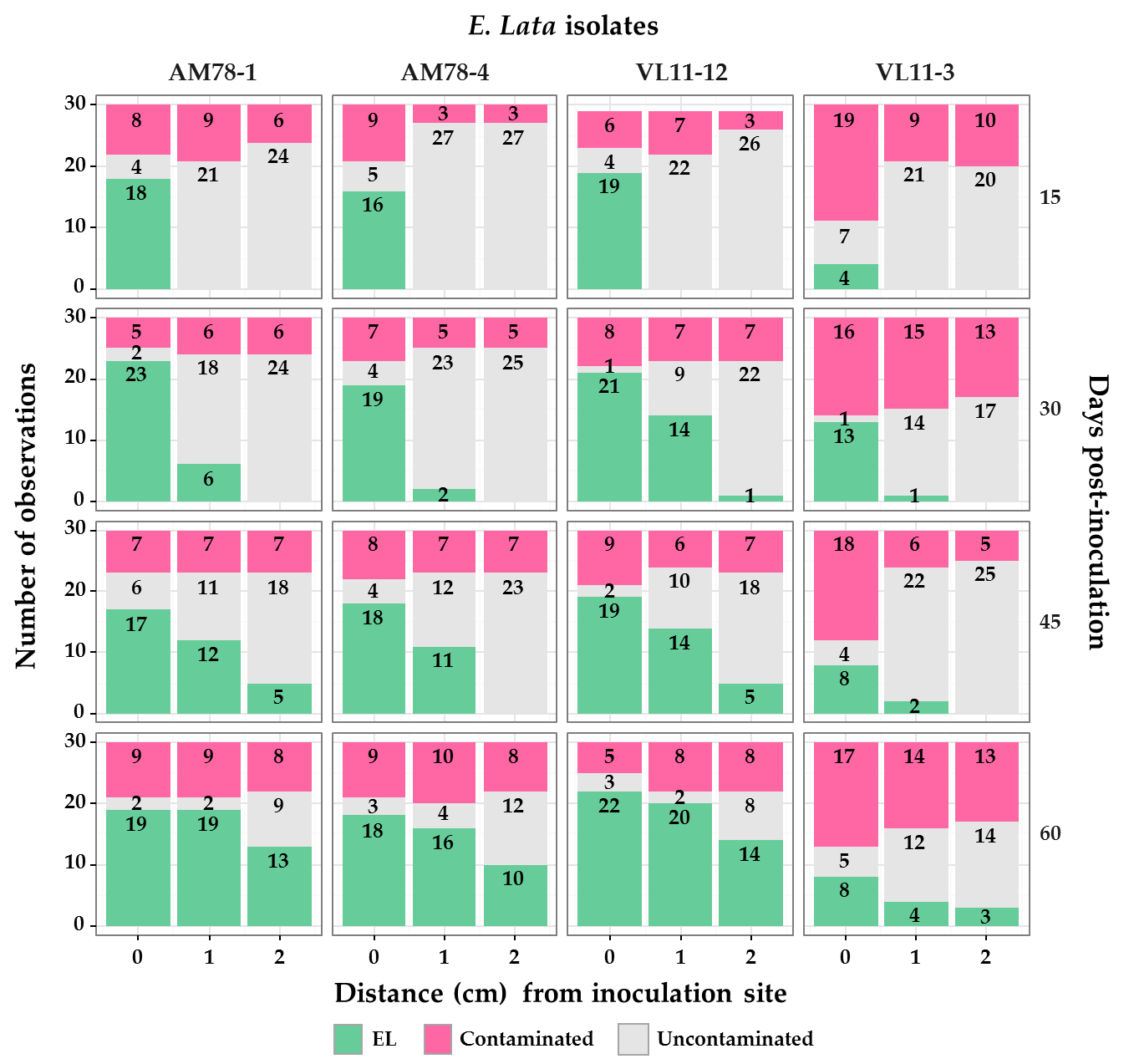


**Figure S1.** **Comparison of four isolates of *E. lata* for their ability to colonize grapevine wood.** Detection of *E. lata* in wood samples, based on the inoculation of 480 cuttings and re-isolations from 1440 wood chips sampled at 0, 1 and 2 cm from the inoculation point. Legend: “EL”= presence of *E. lata* in the sample (in green); “Uncontaminated”= *E. lata* not detected (in grey); “contaminated”= sample with undetermined fungal contamination (in red).


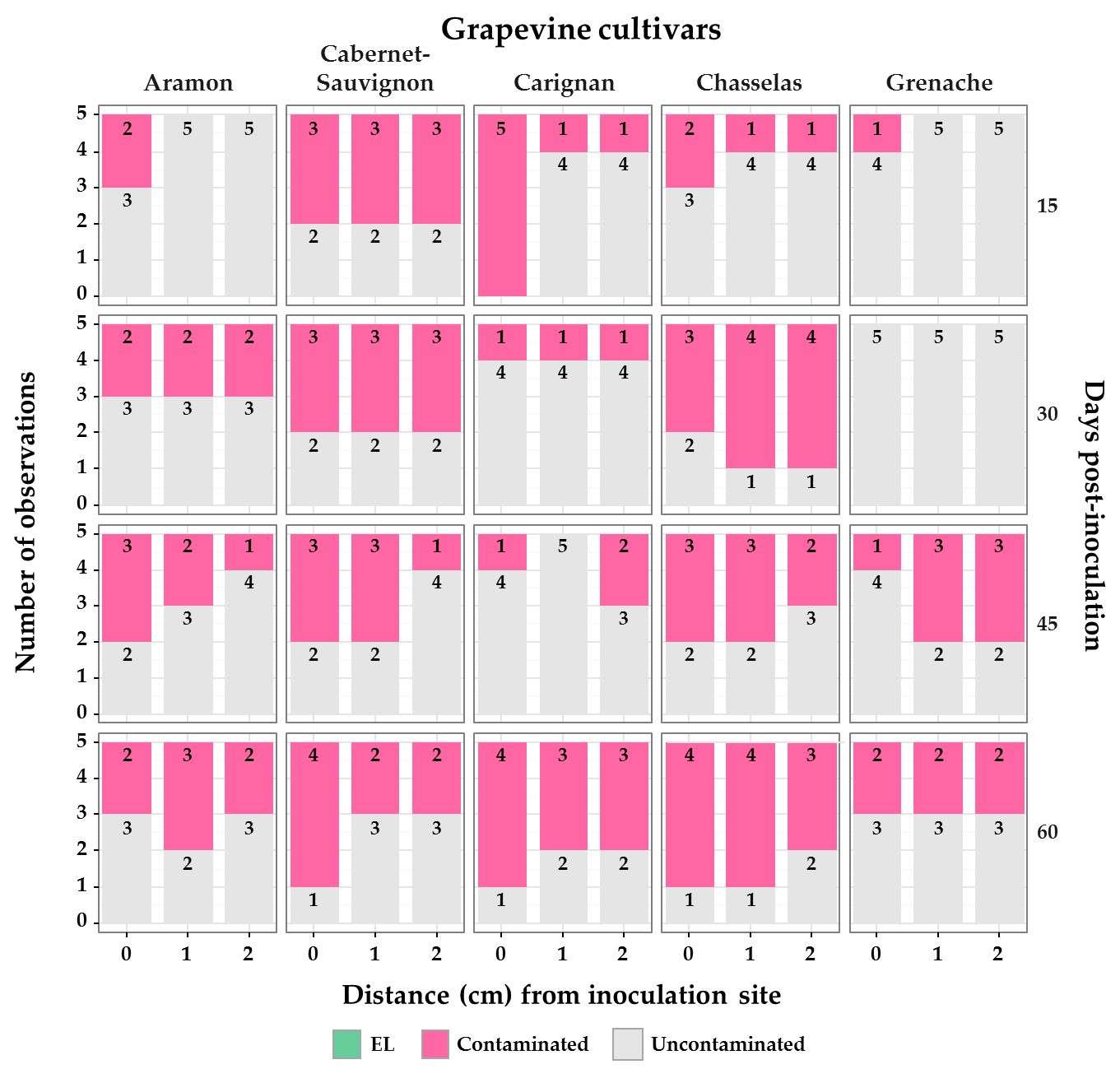


**Figure S2.** **Contaminations observed in wood chips sampled in control plants.** Pathogen re-isolation was performed for 400 wood samples collected on 100 control cuttings (20 cuttings per cultivars), inoculated with plugs of sterile PDA medium. Wood samples were collected at 0, 1 and 2 cm from the inoculation point. Legend: see Figure S1.

**
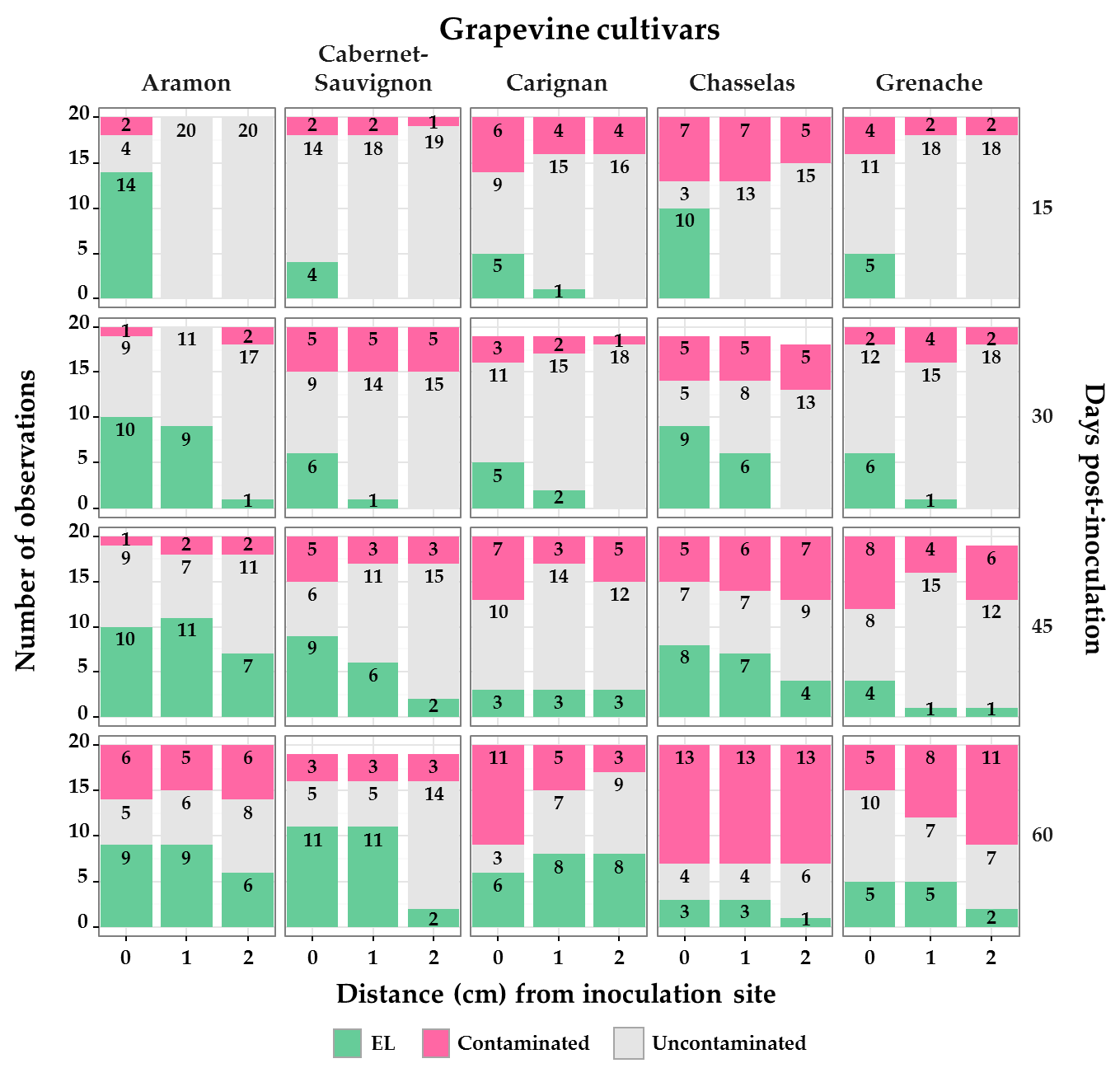
**

**Figure S3.** **Comparison of grapevine cultivars for their tolerance to wood colonization by *E. lata* and other microorganisms.** Pathogen re-isolation was performed for 1200 wood chips sampled on 400 cuttings (20 cuttings per cultivars) inoculated with isolate VL11-12. Wood samples were collected at 0, 1 and 2 cm from the inoculation point. Legend: see Figure S1.


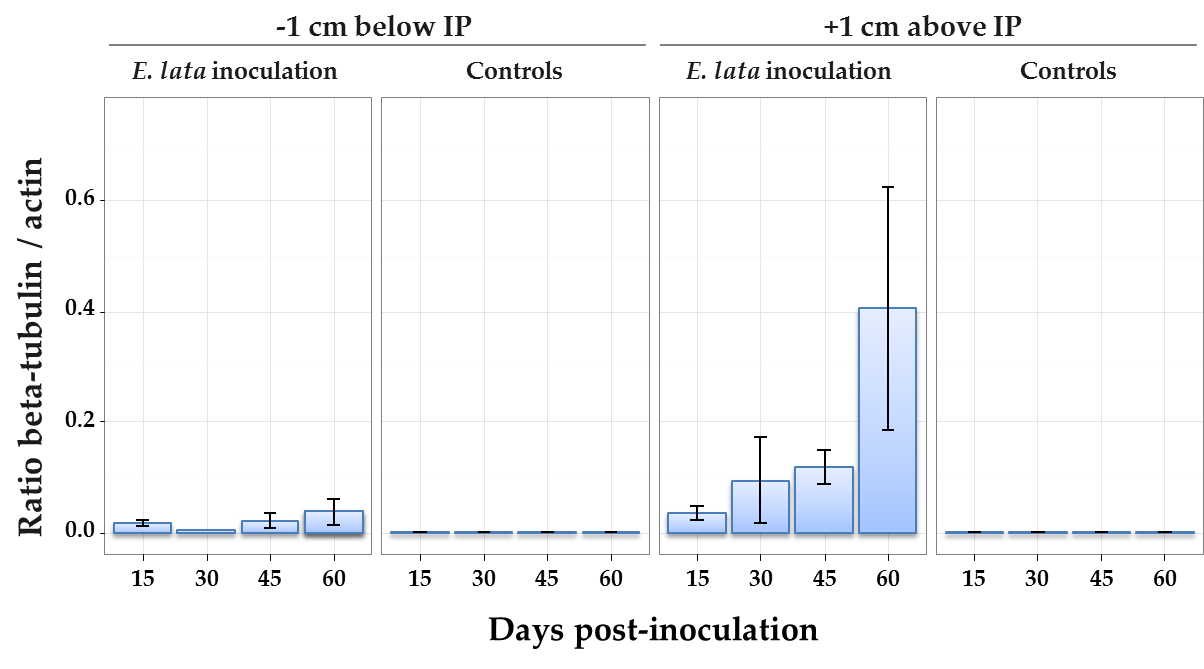


**Figure S4.** **Wood colonization by *E. lata* monitored by qRT-PCR: ratio β-tubulin / actin.** Samples collected at 1 cm above and below the IP, at different days after inoculation. Legend on top, distance from IP (1 cm below or above), and treatment (inoculation with *E. lata*, or with sterile PDA medium for controls). Standard error bars indicate range of values for biological and technical replicates.

© 2017 by Moisy *et al*. Submitted for possible open access publication under the
terms and conditions of the Creative Commons Attribution (CC BY) license (http://creativecommons.org/licenses/by/4.0/).
