# Supplementary material for: Quantitative Assessment of Grapevine Wood Colonization by the Dieback Fungus Eutypa lata
Source: J Fungi (Basel). 2017 May 6;3(2):21. doi: 10.3390/jof3020021 (PMC5715921; doi:10.3390/jof3020021)
Supplement: Supplementary file 1 [file jof-03-00021-s001.zip › Analysis_and_Figures_for_Manuscript.zip!/Analysis_and_Figures_for_Manuscript.pdf]

ANALYSIS AND FIGURES FOR MANUSCRIPT

Author: MOISY C.

08/03/2017

Contents

1 Preamble **2**
1.1 R Markdown document . . . 2
1.2 Working directory . . . 2

2 Load data **3**
2.1 Experiment 1 . . . 3
2.2 Experiment 2 . . . 3
2.3 Experiment 3 . . . 4
2.4 Experiment 4 . . . 4

3 Load functions **5**
3.1 For means, percentages and counts . . . 5
3.2 For standard errors . . . 6

4 Experiment 1 **6**
4.1 Figure S1 . . . 6
4.2 Figure 1 . . . 7
4.2.1 Select and format the data . . . 7
4.2.2 Statistical (proportion) tests . . . 8
4.2.2.1 for AM isolates . . . 8
4.2.2.2 for VL isolates . . . 9
4.2.3 Plot Figure 1 . . . 11
4.3 Figure 2 . . . 12
4.3.1 Means and percentages . . . 12
4.3.2 Plot . . . 13

5 Experiment 2 **15**
5.1 Figure S2 . . . 15
5.2 Figure S3 . . . 16
5.3 Figure 3 . . . 17
5.3.1 Inoculation success percentages . . . 17
5.3.2 Proportion tests . . . 18
5.3.3 Plot . . . 19
5.4 Figure 4 . . . 21

6 Experiment 3 **23**
6.1 Figure 5 . . . 23
6.2 Figure S4 . . . 25
6.3 Analysis (figure 5) . . . 27
6.3.1 Set up dataframe . . . 27
6.3.2 Explore data (interaction plot) . . . 28
6.3.3 Linear model fit . . . 28
6.3.4 Diagnostics via residuals . . . 28
6.3.5 Anova . . . 29

7 Experiment 4 **30**
7.1 Figure 6 . . . 30
7.2 Analysis (figure 6) . . . 31
7.2.1 Set up dataframe . . . 31
7.2.2 Explore data (interaction plot) . . . 32
7.2.3 Linear model fit . . . 32
7.2.4 Diagnostics via residuals . . . 32
7.2.5 Anova . . . 33

This document is a supplementary data from the manuscript "Quantitative assessment of grapevine wood colonization by the dieback fungus *Eutypa lata*" submitted to Journal of Fungi in 2017.

Cédric Moisy (1), Gilles Berger (2), Timothée Flutre (2), Loïc Le Cunff (1) and Jean-Pierre Péros (2)
(1) Institut Français de la Vigne et du Vin, UMT Géno-Vigne, F-34060 Montpellier, France
(2) INRA, UMR AGAP, F-34060 Montpellier, France

License: none

IMPORTANT NOTE:
In order to run this code on your own computer:
1- create a folder somewhere,
2- copy the ".Rmd" file in this folder,
3- copy all the data files in the same folder,
4- specify the correct path to this folder in the "working directory" section below,
5- knit the ".Rmd" file to convert it into HTML (you may have to install external packages as indicated below).

1 Preamble

1.1 R Markdown document

This is an R Markdown document. Markdown is a simple formatting syntax for authoring HTML, PDF, and MS Word documents. For more details on using R Markdown see http://rmarkdown.rstudio.com. When you click the **Knit** button a document will be generated that includes both content as well as the output of any embedded R code chunks within the document.

1.2 Working directory

In order to convert the Rmd file into HTML and PDF while reproducing the whole analysis, we need to specify the path to the directory containing all necessary files.

```
########## YOU NEED TO SPECIFY THIS PATH #################
# data.dir <- "PATH" <<<<<< ! ! !
############## ########## ########## ########## ##########
stopifnot(file.exists(data.dir))
```

You also need external packages:

Note that "The Bayesian First Aid" package requires a working installation of JAGS.
Bååth, R., (2014) Bayesian First Aid: A Package that Implements Bayesian Alternatives to the Classical *.test Functions in R. In the proceedings of UseR! 2014 - the International R User Conference (pdf). To install the package from github you also need the devtools package.

2 Load data

2.1 Experiment 1

Wood colonization in relation to aggressiveness and distance from inoculation site

```
d1 <- "EL_wood_compare_4_isolates.txt"
data1 <- read.table(file=paste0(data.dir, "/", d1), skip=4, header=TRUE, sep="\t", quote="")
head(data1)
```

```
##   n  date dpi    treat                var rep dist_cm EL
## 1 1 01/08  15 VL_11-12 Cabernet-Sauvignon   1       0  0
## 2 2 01/08  15 VL_11-12 Cabernet-Sauvignon   2       0  1
## 3 3 01/08  15 VL_11-12 Cabernet-Sauvignon   3       0  1
## 4 4 01/08  15 VL_11-12 Cabernet-Sauvignon   4       0  C
## 5 5 01/08  15 VL_11-12 Cabernet-Sauvignon   5       0  0
## 6 6 01/08  15 VL_11-12 Cabernet-Sauvignon   6       0  1
```

```
summary(data1)
```

```
##        n            date          dpi            treat
##  Min.   :   1   01/08:360   Min.   :15.0   AM_78-1 :360
##  1st Qu.: 361   10/08:360   1st Qu.:26.2   AM_78-4 :360
##  Median : 720   12/09:360   Median :37.5   VL_11-12:360
##  Mean   : 720   24/08:360   Mean   :37.5   VL_11-3 :360
##  3rd Qu.:1080               3rd Qu.:48.8
##  Max.   :1440               Max.   :60.0
##                  var            rep          dist_cm      EL
##  Cabernet-Sauvignon:1440   Min.   : 1.0   Min.   :0   0   :599
##                            1st Qu.: 8.0   1st Qu.:0   1   :434
##                            Median :15.5   Median :1   C   :404
##                            Mean   :15.5   Mean   :1   NA's:  3
##                            3rd Qu.:23.0   3rd Qu.:2
##                            Max.   :30.0   Max.   :2
```

2.2 Experiment 2

Comparison of grapevine cultivars for their tolerance wood colonization by *E. lata*.

```
d2 <- "EL_wood_compare_5_cultivars_Vv.txt"
data2 <- read.table(file=paste0(data.dir, "/", d2), skip=4, header=TRUE, sep="\t", quote="")
head(data2)
```

```
##   n    date dpi treat    var rep dist_cm EL
## 1 1 08-août  15    T- Aramon   1       0  C
## 2 2 08-août  15    T- Aramon   2       0  0
## 3 3 08-août  15    T- Aramon   3       0  0
## 4 4 08-août  15    T- Aramon   4       0  0
## 5 5 08-août  15    T- Aramon   5       0  C
## 6 6 08-août  15    T- Aramon   1       1  0
```

```
summary(data2)
```

```
##        n              date          dpi              treat
##  Min.   :   1   05-sept:375   Min.   :15.0   T-      : 300
##  1st Qu.: 376   08-août:375   1st Qu.:26.2   VL_11-12:1200
```

```
##  Median : 750   19-sept:375   Median :37.5
##  Mean   : 750   22-août:375   Mean   :37.5
##  3rd Qu.:1125                 3rd Qu.:48.8
##  Max.   :1500                 Max.   :60.0
##                   var            rep          dist_cm      EL
##  Aramon             :300   Min.   : 1   Min.   :0   0   :831
##  Cabernet-Sauvignon:300   1st Qu.: 4   1st Qu.:0   1   :263
##  Carignan           :300   Median : 8   Median :1   C   :395
##  Chasselas          :300   Mean   : 9   Mean   :1   NA's: 11
##  Grenache           :300   3rd Qu.:14   3rd Qu.:2
##                            Max.   :20   Max.   :2
```

2.3 Experiment 3

qPCR as a tool to evaluate wood colonization by *E. lata*.

```
dpi <- "EL_qPCR_different_points.txt"
dpi.df<- read.table(file=paste0(data.dir, "/", dpi), header=TRUE, skip=2, sep="\t")
head(dpi.df)
```

```
##   n          sample treat   PCR dist_cm dpi rep nb_copies
## 1 1 EL_2sem_1cm-_1    EL actin      1-  15   1     18648
## 2 2 EL_2sem_1cm-_2    EL actin      1-  15   2        NA
## 3 3 EL_2sem_1cm-_3    EL actin      1-  15   3     31694
## 4 4 EL_2sem_1cm-_4    EL actin      1-  15   4      7695
## 5 5 EL_4sem_1cm-_1    EL actin      1-  30   1     29207
## 6 6 EL_4sem_1cm-_2    EL actin      1-  30   2     33408
```

```
summary(dpi.df)
```

```
##        n                       sample    treat       PCR     dist_cm
##  Min.   :  1.0   T-_8sem_1cm+_3:  3   EL :64   actin:68   1-  :64
##  1st Qu.: 34.8   EL_2sem_1cm-_1:  2   H2O: 8   EL   :68   1+  :64
##  Median : 68.5   EL_2sem_1cm-_2:  2   T- :64              NA's: 8
##  Mean   : 68.5   EL_2sem_1cm-_3:  2
##  3rd Qu.:102.2   EL_2sem_1cm-_4:  2
##  Max.   :136.0   EL_2sem_1cm+_1:  2
##                  (Other)       :123
##       dpi             rep          nb_copies
##  Min.   :15.0   Min.   :1.00   Min.   :    0
##  1st Qu.:15.0   1st Qu.:2.00   1st Qu.:   18
##  Median :30.0   Median :3.00   Median : 3113
##  Mean   :36.2   Mean   :2.52   Mean   :11437
##  3rd Qu.:45.0   3rd Qu.:4.00   3rd Qu.:22134
##  Max.   :60.0   Max.   :4.00   Max.   :40199
##                                NA's   :20
```

2.4 Experiment 4

Comparison of *E. lata* aggressiveness by qPCR.

```
data.agr <- paste0(data.dir,"/data_AGR.csv")
d.agr <- read.table(file=data.agr, header=TRUE, sep=";")
head(d.agr)
```

```
##   sample Treatment Rep btub_Elata   sd       agr_level stroma
## 1      1   AM 78.1   1      18533  605 high-aggressive     AM
## 2      2   AM 78.1   2      47926  727 high-aggressive     AM
## 3      3   AM 78.1   3      20755 1519 high-aggressive     AM
## 4      4   AM 78.4   1       9323  199  low-aggressive     AM
## 5      5   AM 78.4   2      12379 1498  low-aggressive     AM
## 6      6   AM 78.4   3      16267  384  low-aggressive     AM
```

```
summary(d.agr)
```

```
##      sample      Treatment      Rep      btub_Elata          sd
##  Min.   : 1   AM 78.1 :3   Min.   :1   Min.   :    7   Min.   :   0
##  1st Qu.: 6   AM 78.4 :3   1st Qu.:1   1st Qu.: 5311   1st Qu.: 244
##  Median :11   BX 1.10 :3   Median :2   Median :13435   Median : 456
##  Mean   :11   BX 1.5  :3   Mean   :2   Mean   :13828   Mean   : 630
##  3rd Qu.:16   CM 96.07:3   3rd Qu.:3   3rd Qu.:18533   3rd Qu.: 848
##  Max.   :21   CM 96.6 :3   Max.   :3   Max.   :47926   Max.   :1882
##               Control :3
##            agr_level  stroma
##  high-aggressive:9   AM  :6
##  low-aggressive :9   BX  :6
##  NA's           :3   CM  :6
##                      NA's:3
##
##
##
```

3 Load functions

3.1 For means, percentages and counts

```
# Function to calculate the mean and the standard deviation and percentage (NA removed)
# for each group
# data : a data frame
# varname : the name of a column containing the variable to be summarized
# groupnames : vector of column names to be used as grouping variables

data_summary <- function(data, varname, groupnames){
  require(plyr)
  summary_func <- function(x, col){
                c(mean = mean(x[[col]], na.rm=TRUE),
                  sd = sd(x[[col]], na.rm=TRUE),
                  numb = sum(x[[col]], na.rm=TRUE),
                  perc = round(sum(x[[col]], na.rm=TRUE)
                               /length(na.omit(x[[col]]))*100,
                               digits = 0),
                  count.no.NA = length(na.omit(x[[col]]))
                  )
  }
  data_sum <- ddply(data, groupnames, .fun=summary_func, varname)
  #data_sum <- rename(data_sum, c("mean" = varname))
 return(data_sum)
}
```

3.2 For standard errors

```
# Function to calculate the standard error from dataframe x
  std.error <- function(x, na.rm = T) {
      sqrt(var(x, na.rm = na.rm)/length(x[complete.cases(x)]))
  }
```

4 Experiment 1

Comparison of four isolates of *E. lata* for their ability to colonize the wood of grapevine

4.1 Figure S1

```
# load data
  df<- data1

# update labelling in the dataframe
  levels(df$EL)<- c("UNCONTAMINATED","EL","CONTAMINATED")
  df$EL <- factor(df$EL, levels = c("EL","UNCONTAMINATED","CONTAMINATED"))

# barplot
  p <- ggplot(data=na.omit(df), aes(dist_cm, fill=as.factor(EL))) + geom_bar()
  p <- p + stat_count(aes(label = ..count..), geom="text", color="black", vjust=1.1)
  p <- p + xlab("Distance (cm) from inoculation site")
  p <- p + ylab("Number of observations")
  p <- p + scale_fill_manual(values=c("#66CC99", "grey90","#FF67A4"))
  p <- p + facet_grid(dpi ~ treat)
  p <- p + theme_bw() + theme(legend.position="bottom")
  print(p)
```

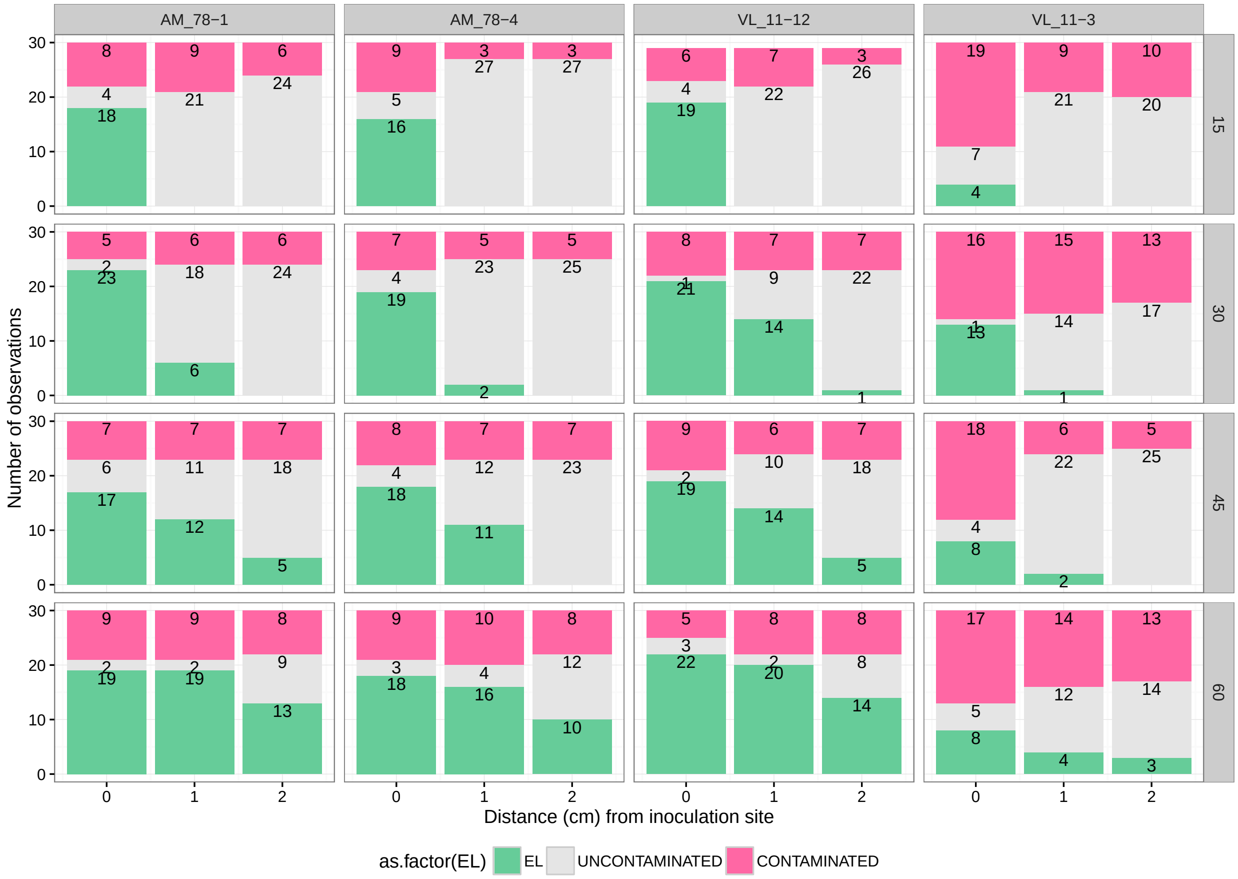

4.2 Figure 1

Comparison of four *E. lata isolates* for their inoculation efficiency

4.2.1 Select and format the data

```
# remove controls (T-)
  d1.noT <- subset(data1, treat!="T-")

# replace contaminated sample by NA
  d1.noT$EL[d1.noT$EL == "C"] <- NA

# formatting
  d1.noT$EL <- as.numeric(as.character(d1.noT$EL))  # as numeric (0/1/NA)

# calculate means and the std.err and percentage
  d1.noT_sum <- data_summary(data= d1.noT, varname= "EL", groupnames=c("treat"))

# keep only values at 0 cm to estimate the inoculation success
  d1.noT.0 <- subset(d1.noT, dist_cm =="0")

# calculate means and the std.err and percentage
```

```r
  d1.noT.0_sum <- data_summary(data= d1.noT.0, varname= "EL", groupnames=c("treat"))

  d1.noT.0_sum$treat.count <- paste0(d1.noT.0_sum$treat, "\n(n= ",
                                      d1.noT.0_sum$count.no.NA,")")

# add column with isolate name
  d1.noT.0_sum$fam <- substr(d1.noT.0_sum$treat, 1, 2)
```

4.2.2 Statistical (proportion) tests

4.2.2.1 for AM isolates

Analysis of proportions with prop.test() and bayes.prop.test()

```r
# select isolates
  d <- subset(d1.noT.0_sum, fam=="AM")

# test proportions with prop.test()
  res.AM.1 <- prop.test(d$numb, d$count.no.NA)
  res.AM.1
```

```
##
##  2-sample test for equality of proportions with continuity
##  correction
##
## data:  d$numb out of d$count.no.NA
## X-squared = 0.1, df = 1, p-value = 0.7
## alternative hypothesis: two.sided
## 95 percent confidence interval:
##  -0.0913  0.1514
## sample estimates:
## prop 1 prop 2
##  0.846  0.816
```

```r
# test proportions with bayes.prop.test()
  res.AM.2 <- BayesianFirstAid::bayes.prop.test(d$numb, d$count.no.NA)
# print results
  res.AM.2
```

```
##
##  Bayesian First Aid proportion test
##
## data: d$numb out of d$count.no.NA
## number of successes:  77, 71
## number of trials:     91, 87
## Estimated relative frequency of success [95% credible interval]:
##   Group 1: 0.84 [0.76, 0.91]
##   Group 2: 0.81 [0.73, 0.89]
## Estimated group difference (Group 1 - Group 2):
##   0.03 [-0.08, 0.14]
## The relative frequency of success is larger for Group 1 by a probability
## of 0.704 and larger for Group 2 by a probability of 0.296 .
```

```r
# plot intervals
  plot(res.AM.2)
```

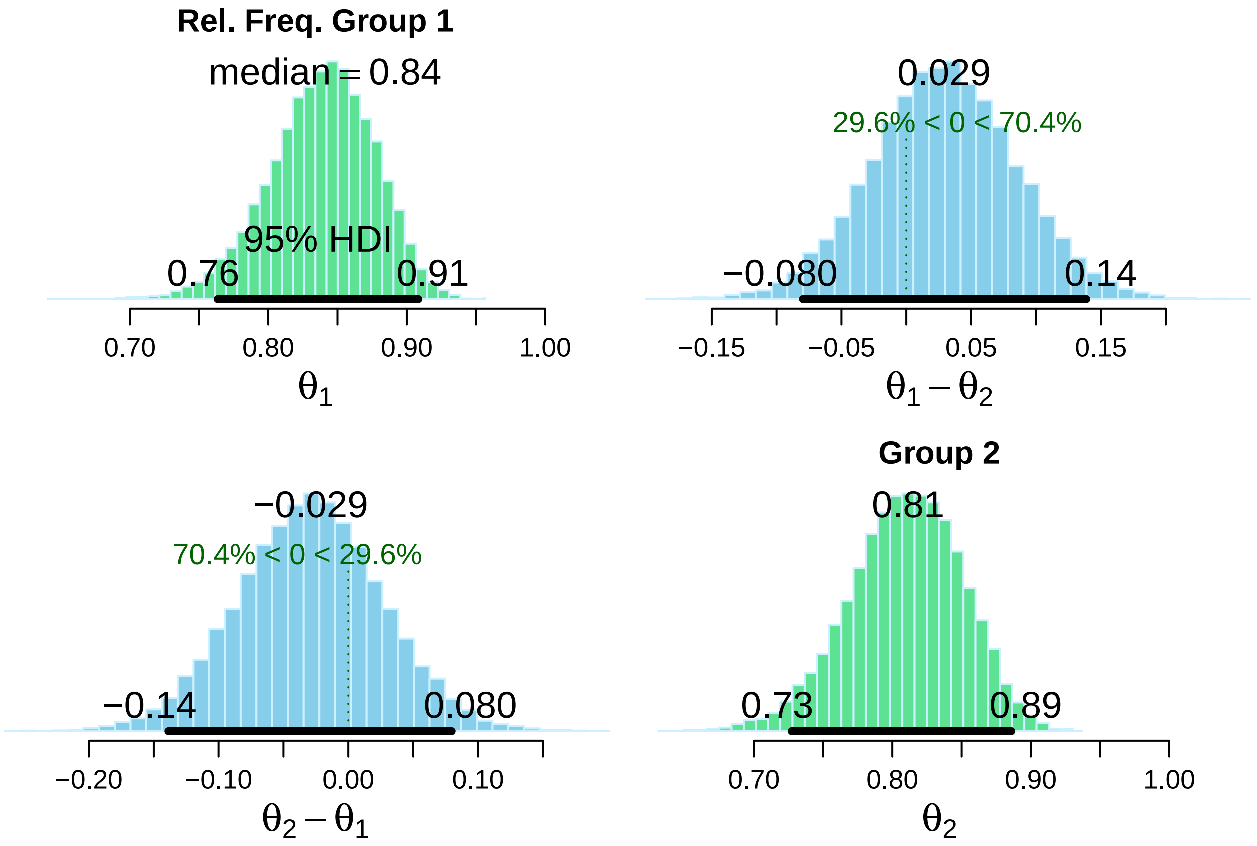

Note that:
Group 1 = AM_78-1
Group 2 = AM_78-4

The estimated relative frequency of inoculation success was not significantly different between isolates AM78-4 and AM78-1.

4.2.2.2 for VL isolates

Analysis of proportions with prop.test() and bayes.prop.test()

```
# select isolates
  d <- subset(d1.noT.0_sum, fam=="VL")

# test proportions with prop.test()
  res.VL.1 <- prop.test(d$numb, d$count.no.NA)
  res.VL.1
```

```
##
##  2-sample test for equality of proportions with continuity
##  correction
##
## data:  d$numb out of d$count.no.NA
## X-squared = 10, df = 1, p-value = 0.002
## alternative hypothesis: two.sided
## 95 percent confidence interval:
##  0.0684 0.3918
## sample estimates:
```

```
## prop 1 prop 2
##   0.89   0.66
```

```r
# test proportions with bayes.prop.test()
  res.VL.2 <- BayesianFirstAid::bayes.prop.test(d$numb, d$count.no.NA)
# print results
  res.VL.2
```

```
##
##  Bayesian First Aid proportion test
##
## data: d$numb out of d$count.no.NA
## number of successes:  81, 33
## number of trials:     91, 50
## Estimated relative frequency of success [95% credible interval]:
##   Group 1: 0.88 [0.82, 0.94]
##   Group 2: 0.66 [0.53, 0.79]
## Estimated group difference (Group 1 - Group 2):
##   0.23 [0.085, 0.37]
## The relative frequency of success is larger for Group 1 by a probability
## of >0.999 and larger for Group 2 by a probability of <0.001 .
```

```r
# plot intervals
  plot(res.VL.2)
```

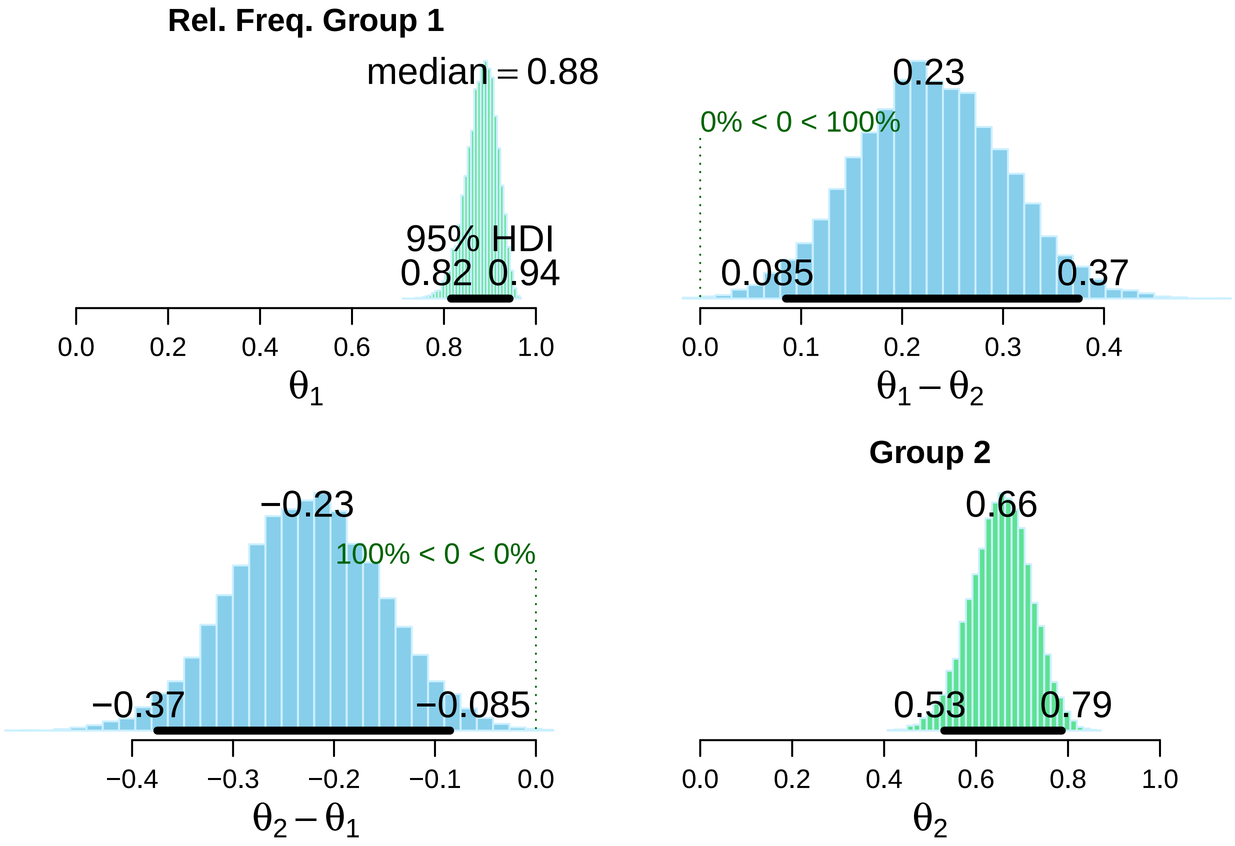

Note that:
Group 1 = VL_11-12
Group 2 = VL_11-3

The estimated relative frequency of inoculation success was significantly different between isolates VL11-3 and VL11-12 by a probability of 99.9 %.

4.2.3 Plot Figure 1

```
# estimate credible intervals
  result.test.1 <- BayesianFirstAid::bayes.prop.test(d1.noT.0_sum$numb,
                                                     d1.noT.0_sum$count.no.NA)

# The object "result.test.1$stats"" contains the 95-percent-credible intervals
# in the "HDIlo" & "HDIup" in columns ([1:5,5:6]).
# We need to retrieve them to set up the std error bars in the next plot
  res <- as.data.frame(result.test.1$stats)
  lim.down <- res[1:4,5]
  lim.up   <- res[1:4,6]

# Define the top and bottom of the errorbars
  limits <- aes(ymax= lim.up *100,
                ymin= lim.down *100)

# set colors
   my_col=c("brown","palegreen", "brown","palegreen")

# barplot and std.error
  p <- ggplot(d1.noT.0_sum, aes(as.factor(treat.count), perc, fill=treat))
  p <- p + geom_bar(stat="identity", position="dodge", color="black")
  p <- p + geom_errorbar(limits, position=position_dodge(.9),width=.2)
  p <- p + geom_text(aes(label=paste0(round(perc,0)," %")),
                    position=position_dodge(width=0.9), vjust=-.5)
  p <- p + scale_fill_manual(values= my_col)
  p <- p + ylim(0, 100)
  p <- p + labs(title= "Figure 1",
                x= "EL isolates",
                y= "Inoculation efficiency (%)")
  p <- p + facet_grid(. ~ fam, scales = "free")
  p <- p + theme_bw()  + theme(legend.position="bottom")
print(p)
```

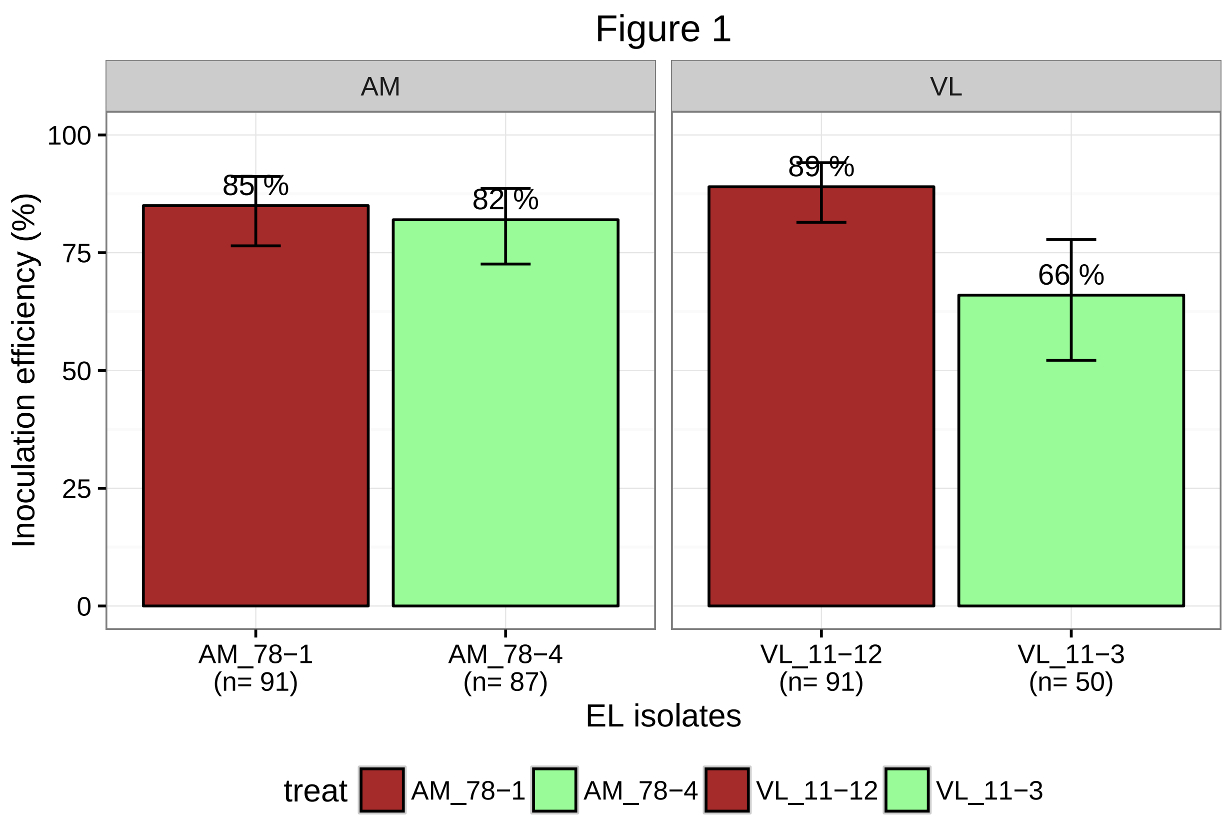

95% credible intervals were calculated for each cultivar using Bayesian proportion test and represented as error bars in the figure.

4.3 Figure 2

Percentage of wood samples colonized by four *E. lata* isolates

4.3.1 Means and percentages

Calculate the means, percentages and counts

```
# Replace contaminated sample "C" by "NA" in the dataframe
  data1$EL[data1$EL == "C"] <- NA

# formatting
  data1$EL <- as.numeric(as.character(data1$EL))         # as numeric (0/1/NA)
  data1$dpi <- as.factor(as.character(data1$dpi))        # as numeric (15/30/45/60)
  data1$dist_cm <- as.factor(as.character(data1$dist_cm))# as numeric (0/1/2)

# means, percentages and counts
  data1_sum <- data_summary(data= data1, varname= "EL",
                          groupnames=c("dpi","treat","var","dist_cm"))
# update column names
  colnames(data1_sum)[5]<- "EL.mean"
  colnames(data1_sum)[6]<- "EL.mean.sd"
  colnames(data1_sum)[8]<- "EL.perc"
```

```r
head(data1_sum)
```

```
##   dpi   treat                var dist_cm EL.mean EL.mean.sd numb EL.perc
## 1  15 AM_78-1 Cabernet-Sauvignon       0   0.818      0.395   18      82
## 2  15 AM_78-1 Cabernet-Sauvignon       1   0.000      0.000    0       0
## 3  15 AM_78-1 Cabernet-Sauvignon       2   0.000      0.000    0       0
## 4  15 AM_78-4 Cabernet-Sauvignon       0   0.762      0.436   16      76
## 5  15 AM_78-4 Cabernet-Sauvignon       1   0.000      0.000    0       0
## 6  15 AM_78-4 Cabernet-Sauvignon       2   0.000      0.000    0       0
##   count.no.NA
## 1          22
## 2          21
## 3          24
## 4          21
## 5          27
## 6          27
```

```r
str(data1_sum)
```

```
## 'data.frame':    48 obs. of  9 variables:
##  $ dpi        : Factor w/ 4 levels "15","30","45",..: 1 1 1 1 1 1 1 1 1 1 ...
##  $ treat      : Factor w/ 4 levels "AM_78-1","AM_78-4",..: 1 1 1 2 2 2 3 3 3 4 ...
##  $ var        : Factor w/ 1 level "Cabernet-Sauvignon": 1 1 1 1 1 1 1 1 1 1 ...
##  $ dist_cm    : Factor w/ 3 levels "0","1","2": 1 2 3 1 2 3 1 2 3 1 ...
##  $ EL.mean    : num  0.818 0 0 0.762 0 ...
##  $ EL.mean.sd : num  0.395 0 0 0.436 0 ...
##  $ numb       : num  18 0 0 16 0 0 19 0 0 4 ...
##  $ EL.perc    : num  82 0 0 76 0 0 83 0 0 36 ...
##  $ count.no.NA: num  22 21 24 21 27 27 23 22 26 11 ...
```

4.3.2 Plot

Plot Figure 2: Percentage of wood samples colonized by four *E. lata* isolates

```r
# add label
  data1_sum$mylabel <- paste0(data1_sum$EL.perc,"%(n=",data1_sum$count.no.NA,")")

### FUNCTION to compare EL aggressivene ss,

ggplot.comp.aggr.by.dist <- function(d, distance, main){
  # d= dataframe with "dpi", "EL.perc", "dist_cm" columns
  # fungus= name of the fungus considered, needed to subset data

  # subset data for the variety
    d <-subset(d, d$dist_cm==distance)

  # ggplot
  p <- ggplot(d, aes(x=dpi, y=EL.perc, group=treat))
  p <- p +  geom_line(aes(linetype=treat, color=treat, size=treat))
  p <- p +  geom_point(aes(shape=treat), size=3)
  p <- p +  scale_linetype_manual(values=c("dashed","dashed","solid","solid"))
  p <- p +  scale_color_manual(values= c("red","green","brown","green"))
  p <- p +  scale_size_manual(values=c(1,1,1,1))
  p <- p + labs(title = paste0("at ",distance, " cm from IP"))
```

```
  p <- p +  ylim(c(0, 100))
  p <- p +  theme_bw() +  theme(legend.position="bottom")
  print(p)
 }

# remove distance "0 cm" before plotting
  d <- subset(data1_sum, dist_cm!="0")

# plotting
  p1 <- ggplot.comp.aggr.by.dist(d, distance=1, main=my_title)
```

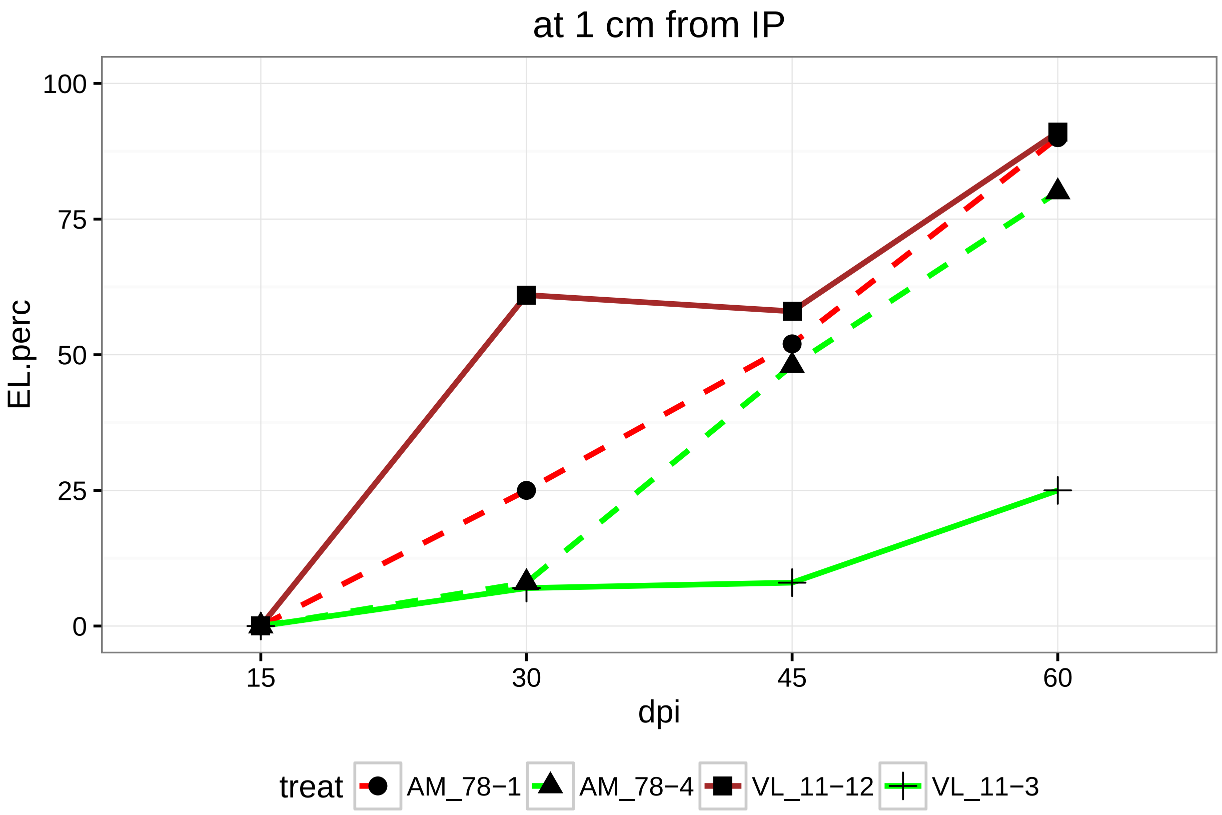


```
  p2 <- ggplot.comp.aggr.by.dist(d, distance=2, main=my_title)
```

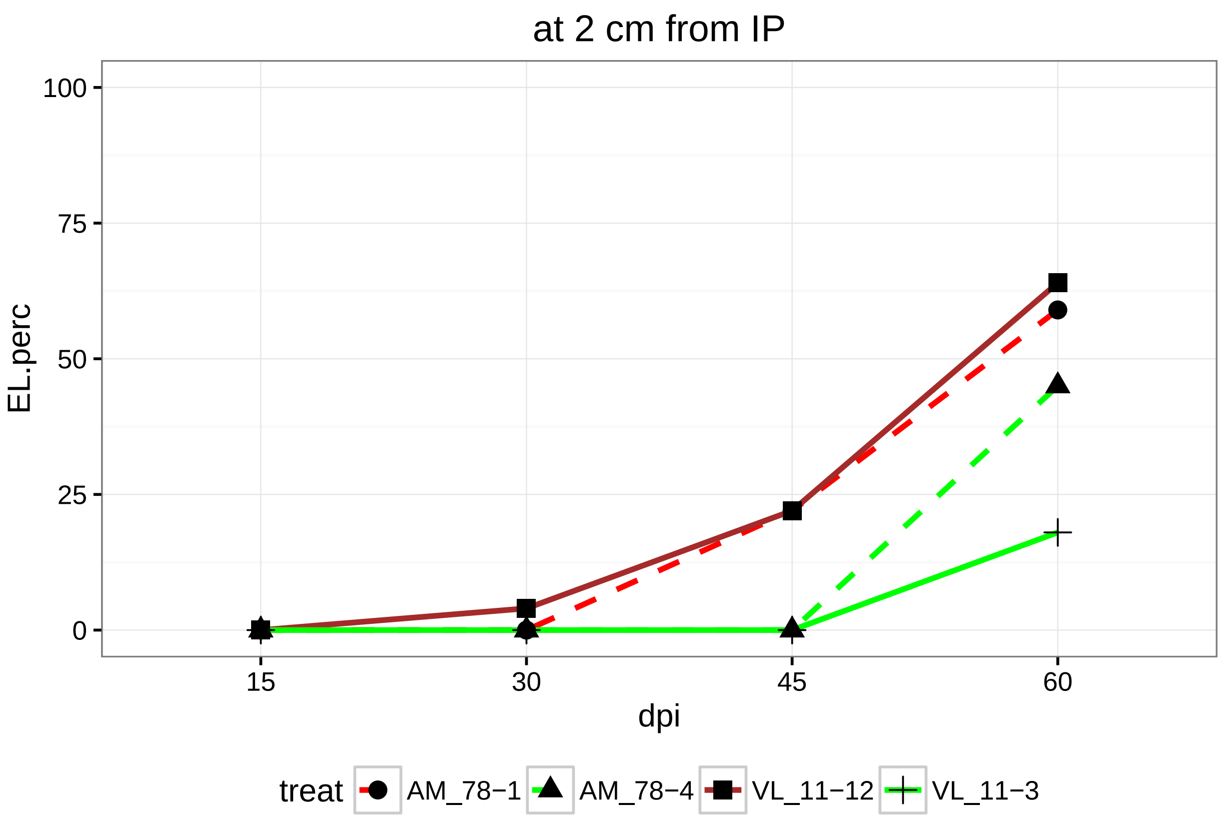

5 Experiment 2

Comparison of five grapevine varieties for their tolerance to *E. lata* colonization

5.1 Figure S2

Contaminations observed in wood chips sampled in control plants.

```
# load data
  df<- data2

# replace "C" by "conta" and !="C" by "uncontam." in df
  levels(df$EL)<- c("UNCONTAMINATED","EL","CONTAMINATED")
  df$EL <- factor(df$EL, levels = c("EL","UNCONTAMINATED","CONTAMINATED"))

# select data from controls
  tmp <- subset(na.omit(df),treat=="T-")
  levels(tmp$treat)[1]<- "CONTROLS"

# plot results
  p <- ggplot(data=tmp, aes(dist_cm, fill=as.factor(EL))) + geom_bar()
  p <- p + stat_count(aes(label = ..count..), geom="text", color="black", vjust=1.1)
  p <- p + xlab("Distance from inoculation site")+ ylab("Number of observations")
  p <- p + scale_fill_manual(values=c("grey90","#FF67A4"))
```

```
  p <- p + facet_grid(dpi ~ var)+ scale_x_discrete(limits=0:2)
  p <- p + theme_bw() + theme(legend.position="bottom")
print(p)
```

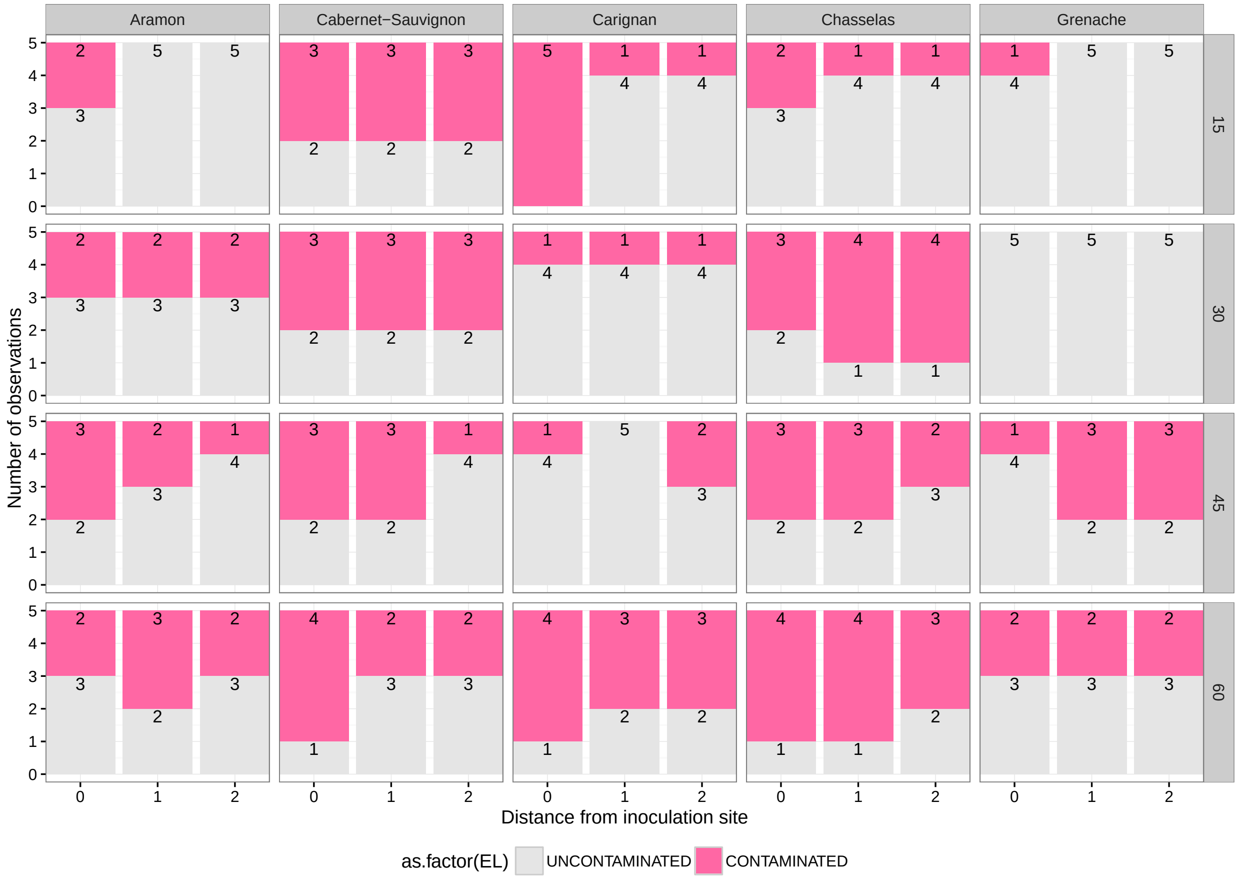

5.2 Figure S3

Comparison of grapevine cultivars for their tolerance to wood colonization by *E. lata* and other microorganisms.

```
# remove controls
  tmp <- subset(na.omit(df),treat!="T-")

# plot results
  p <- ggplot(data=tmp, aes(dist_cm, fill=as.factor(EL))) + geom_bar()
  p <- p + stat_count(aes(label = ..count..), geom="text", color="black", vjust=1.1)
  p <- p + xlab("Distance from inoculation site")+ ylab("Number of observations")
  p <- p + scale_fill_manual(values=c("#66CC99", "grey90","#FF67A4"))
  p <- p + facet_grid(dpi ~ var)+ scale_x_discrete(limits=0:2)
  p <- p + theme_bw() + theme(legend.position="bottom")
print(p)
```

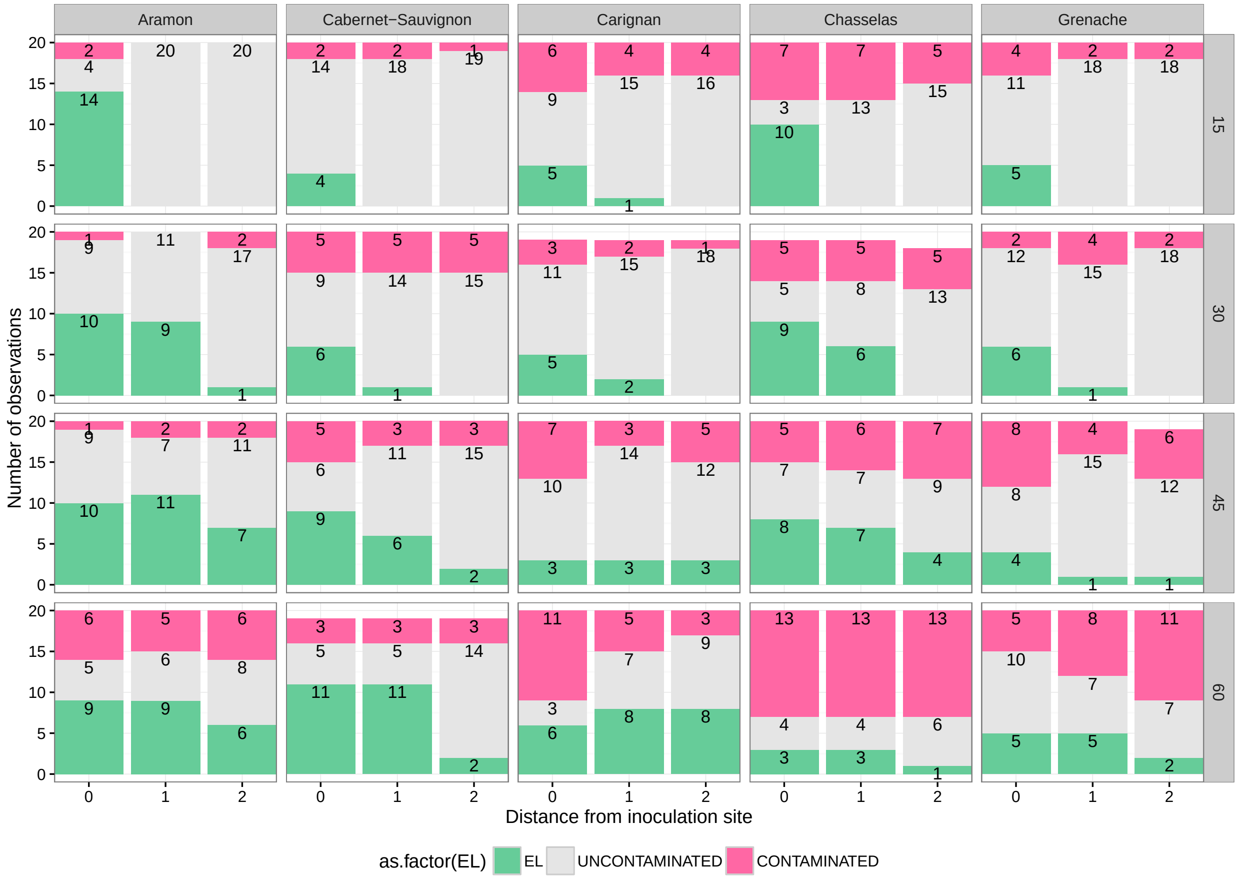

5.3 Figure 3

Comparison of cultivars and proportion tests

5.3.1 Inoculation success percentages

```
# remove controls
  d.noT <- subset(data2, treat!="T-")

# replace contaminated sample by NA
  d.noT$EL[d.noT$EL == "C"] <- NA
# formatting
  d.noT$EL <- as.numeric(as.character(d.noT$EL))  # as numeric (0/1/NA)

# keep only values at 0 cm to illustrate the infection of the cutting
  d.noT.0 <- subset(d.noT, dist_cm=="0")

# calculate means and the std.err and percentage
  d.noT.0_sum <- data_summary(data= d.noT.0, varname= "EL",
                              groupnames=c("var")) # ,"dist_cm","dpi","treat",
```

5.3.2 Proportion tests

Test for equality of proportions with prop.test() and bayes.prop.test() functions.

```r
# check order of cultivars
  d.noT.0_sum$var
```

```
## [1] Aramon             Cabernet-Sauvignon Carignan
## [4] Chasselas          Grenache
## Levels: Aramon Cabernet-Sauvignon Carignan Chasselas Grenache
```

```r
# run test
  result.test.1 <- prop.test(d.noT.0_sum$numb, d.noT.0_sum$count.no.NA)
  result.test.1
```

```
##
##  5-sample test for equality of proportions without continuity
##  correction
##
## data:  d.noT.0_sum$numb out of d.noT.0_sum$count.no.NA
## X-squared = 20, df = 4, p-value = 0.002
## alternative hypothesis: two.sided
## sample estimates:
## prop 1 prop 2 prop 3 prop 4 prop 5
##  0.614  0.469  0.365  0.612  0.328
```

```r
  result.test.2 <- BayesianFirstAid::bayes.prop.test(d.noT.0_sum$numb,
                                                     d.noT.0_sum$count.no.NA)
  result.test.2
```

```
##
##  Bayesian First Aid proportion test
##
## data: d.noT.0_sum$numb out of d.noT.0_sum$count.no.NA
## number of successes:  43, 30, 19, 30, 20
## number of trials:     70, 64, 52, 49, 61
## Estimated relative frequency of success [95% credible interval]:
##   Group 1: 0.61 [0.50, 0.72]
##   Group 2: 0.47 [0.35, 0.59]
##   Group 3: 0.37 [0.25, 0.50]
##   Group 4: 0.61 [0.48, 0.74]
##   Group 5: 0.33 [0.22, 0.45]
## Estimated pairwise group differences (row - column) with 95 % cred. intervals:
##                               Group
##           2               3               4               5
##   1      0.14            0.24             0              0.28
##      [-0.024, 0.3]   [0.082, 0.41]   [-0.17, 0.18]   [0.12, 0.44]
##   2                       0.1            -0.14            0.14
##                     [-0.075, 0.27]  [-0.32, 0.036]   [-0.029, 0.3]
##   3                                      -0.24            0.03
##                                     [-0.42, -0.057]  [-0.13, 0.21]
##   4                                                       0.28
##                                                      [0.093, 0.44]
```

```r
# print and plot bayes.prop.test results
  plot(result.test.2)
```

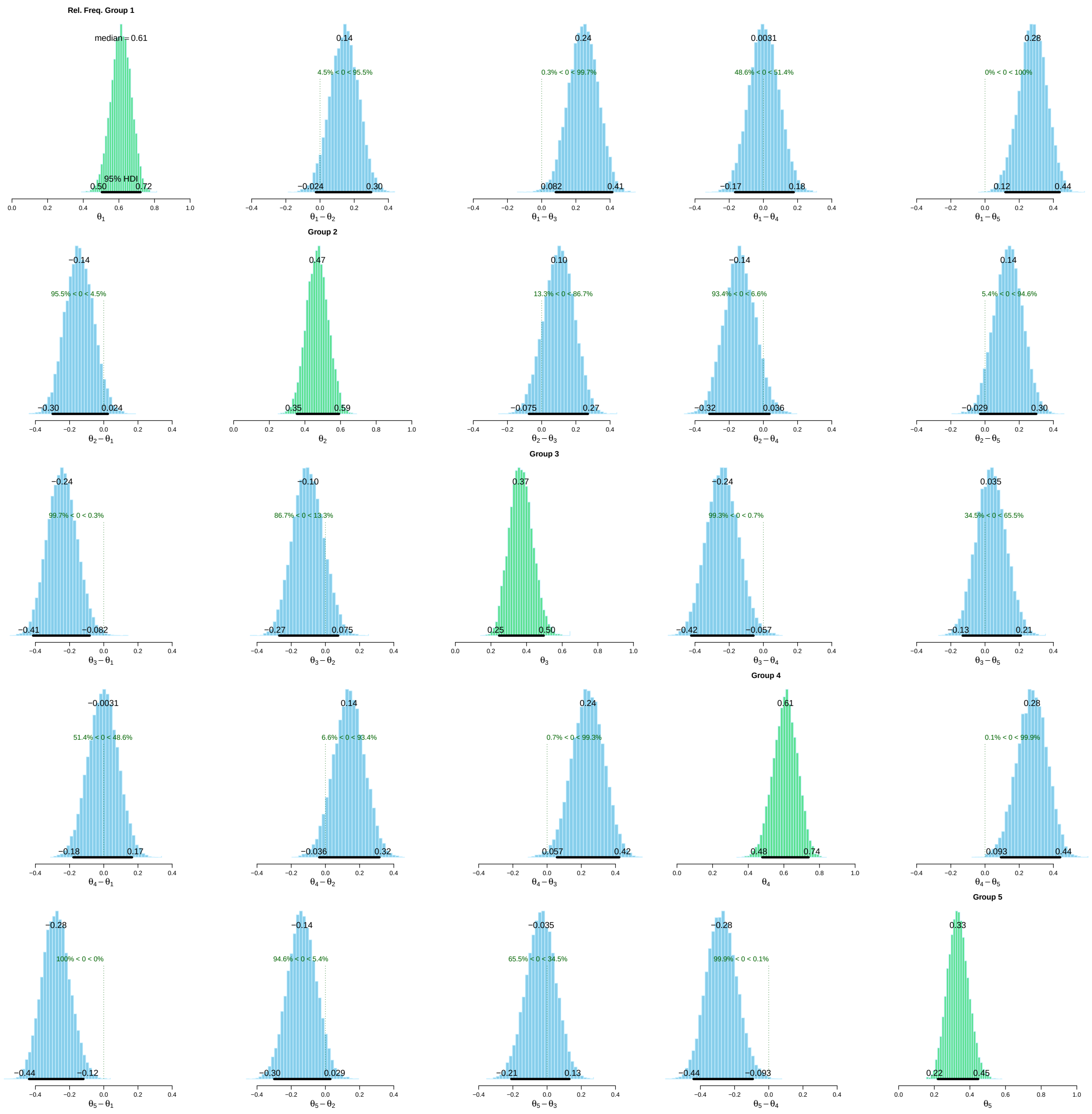

Note that:
Group 1 = Aramon
Group 2 = Cabernet-Sauvignon
Group 3 = Carignan
Group 4 = Chasselas
Group 5 = Grenache

5.3.3 Plot

Figure 3: Comparison of grapevine cultivar on the infection success in cuttings inoculated with *E. lata*.

```
# We need to retrieve the 95-percent-confidence interval and use them
# to set up the std error bars in the next plot
  res <- as.data.frame(result.test.2$stats)
```

```
  lim.down <- res[1:5,5]
  lim.up   <- res[1:5,6]

# Define the top and bottom of the errorbars
  limits <- aes(ymax= lim.up *100,
                ymin= lim.down *100)
# set colors
  my_col=c("grey30","grey50","grey70","grey30","grey90")

# barplot and std.error
  p <- ggplot(d.noT.0_sum, aes(as.factor(reorder(var, perc)), perc, fill=var))
  p <- p + geom_bar(stat="identity", position="dodge", color="black")
  p <- p + geom_errorbar(limits, position=position_dodge(.9),width=.2)
  p <- p + geom_text(aes(label=paste0(round(perc,0)," %\n(n= ",count.no.NA,")")),
                     position=position_dodge(width=0.9), vjust=-.5,
                     color=rep(c("black"),5))
  p <- p + scale_fill_manual(values = my_col)
  p <- p + ylim(0, 75)
  p <- p + labs(x= "Grapevine varieties", y= "Inoculation efficiency (%)")
  p <- p + theme_bw()  + theme(legend.position="bottom")
print(p)
```

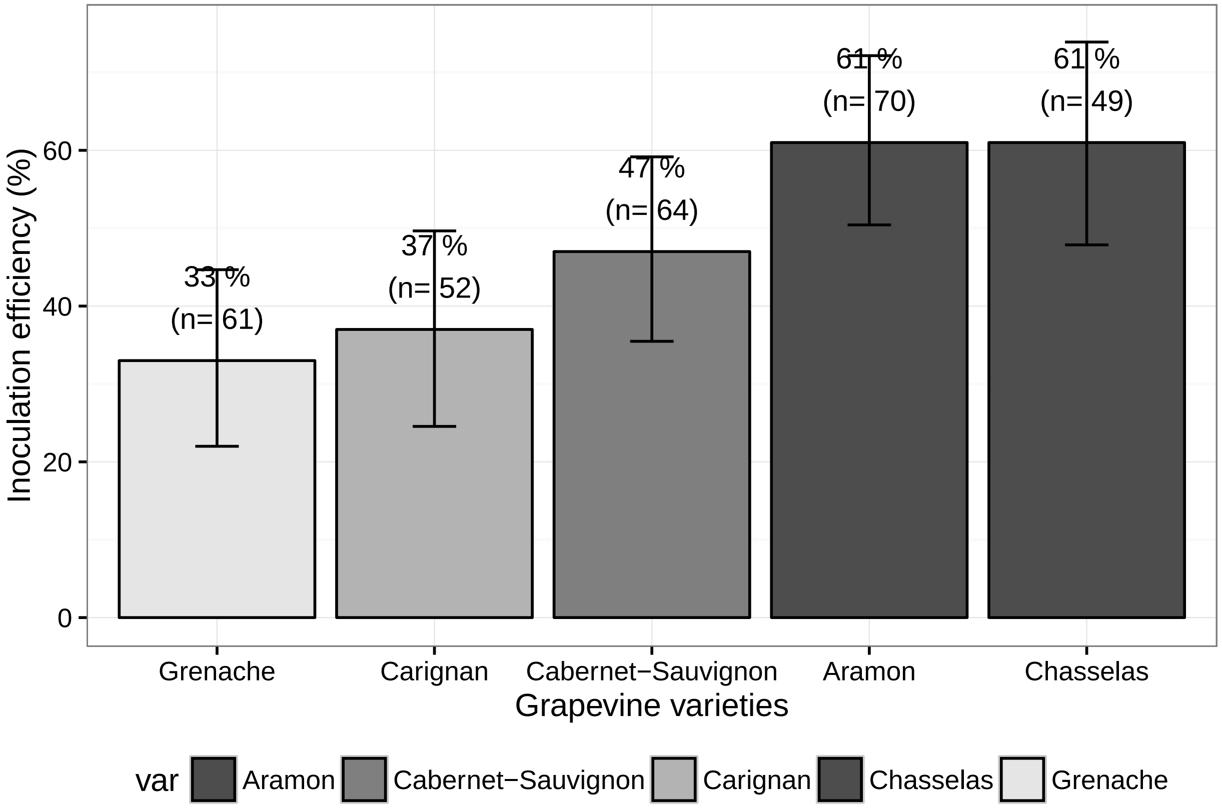

95% credible intervals were calculated for each cultivar using Bayesian proportion test and represented as error bars in the figure.

5.4 Figure 4

Effect of grapevine cultivar on the wood colonization in cuttings inoculated with *E. lata*.

```
# Replace contaminated sample by "NA" in the dataframe
  data2$EL[data2$EL == "C"] <- NA                        # replace "C" by "NA"
  data2$EL <- as.numeric(as.character(data2$EL))         # as numeric (0/1/NA)
  data2$dpi <- as.factor(as.character(data2$dpi))        # as numeric (15/30/45/60)
  data2$dist_cm <- as.factor(as.character(data2$dist_cm))# as numeric (0/1/2)

# calculate the mean and the standard deviation and percentage (NA removed)
  data2_sum <- data_summary(data= data2, varname= "EL",
                        groupnames=c("dpi","treat","var","dist_cm"))
# update column names
  colnames(data2_sum)[5]<- "EL.mean"
  colnames(data2_sum)[6]<- "EL.mean.sd"
  colnames(data2_sum)[8]<- "EL.perc"

# select only inoculations
  d <- subset(data2_sum, data2_sum$treat != "T-")
  d <- subset(d, d$dist_cm != "0")
  head(d)
```

```
##    dpi    treat                var dist_cm EL.mean EL.mean.sd numb EL.perc
## 17  15 VL_11-12             Aramon       1  0.0000       0.00    0       0
## 18  15 VL_11-12             Aramon       2  0.0000       0.00    0       0
## 20  15 VL_11-12 Cabernet-Sauvignon       1  0.0000       0.00    0       0
## 21  15 VL_11-12 Cabernet-Sauvignon       2  0.0000       0.00    0       0
## 23  15 VL_11-12           Carignan       1  0.0625       0.25    1       6
## 24  15 VL_11-12           Carignan       2  0.0000       0.00    0       0
##    count.no.NA
## 17          20
## 18          20
## 20          18
## 21          19
## 23          16
## 24          16
```

```
### FUNCTION to plot colonization of wood per variety

  ggplot.wood.coloniz.per.dist <- function(d, distance){

        dtemp <-subset(d, d$dist_cm==distance)

  # ggplot
  p <- ggplot(dtemp, aes(x=dpi, y=EL.perc, group=var))
  p <- p + geom_line(aes(linetype=var, color=var, size=var))
  p <- p + geom_point(aes(shape=var), size=3)
  p <- p + scale_linetype_manual(values=c("solid","dashed","dashed","solid","solid"))
  p <- p + scale_color_manual(values= c("red","violet","green","brown","blue"))
  p <- p + scale_size_manual(values=c(1,1,1,1,1))
  p <- p + scale_shape_manual(values=c(17,3,15,19,19))
  p <- p + labs(title = paste0("at ",distance, " cm from IP"))
  p <- p + ylim(c(0, 100))
  p <- p + theme_bw() +  theme(legend.position="bottom")
```

```
  print(p)
}

p1 <- ggplot.wood.coloniz.per.dist(d, distance=1)
```

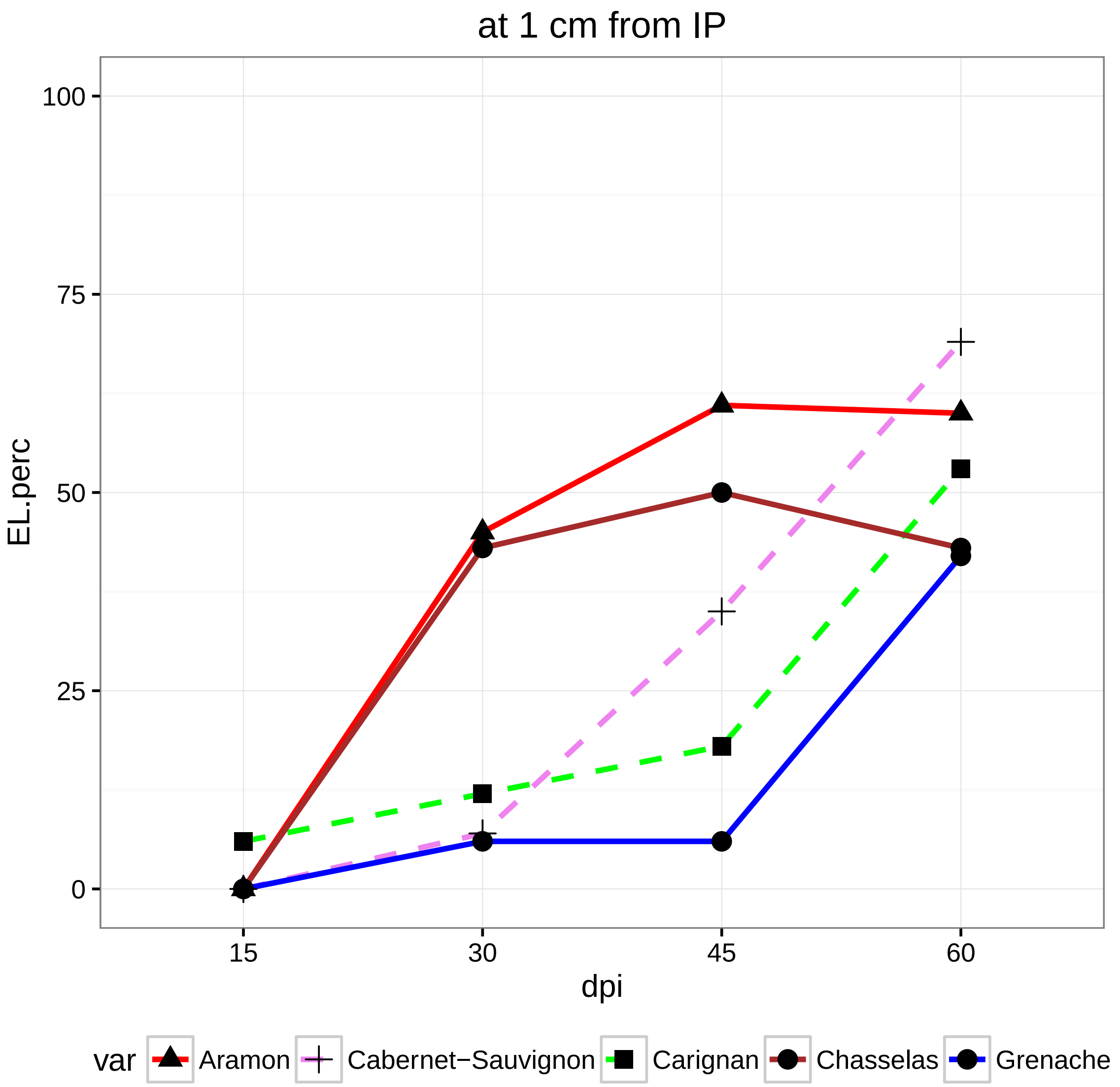


```
p2 <- ggplot.wood.coloniz.per.dist(d, distance=2)
```

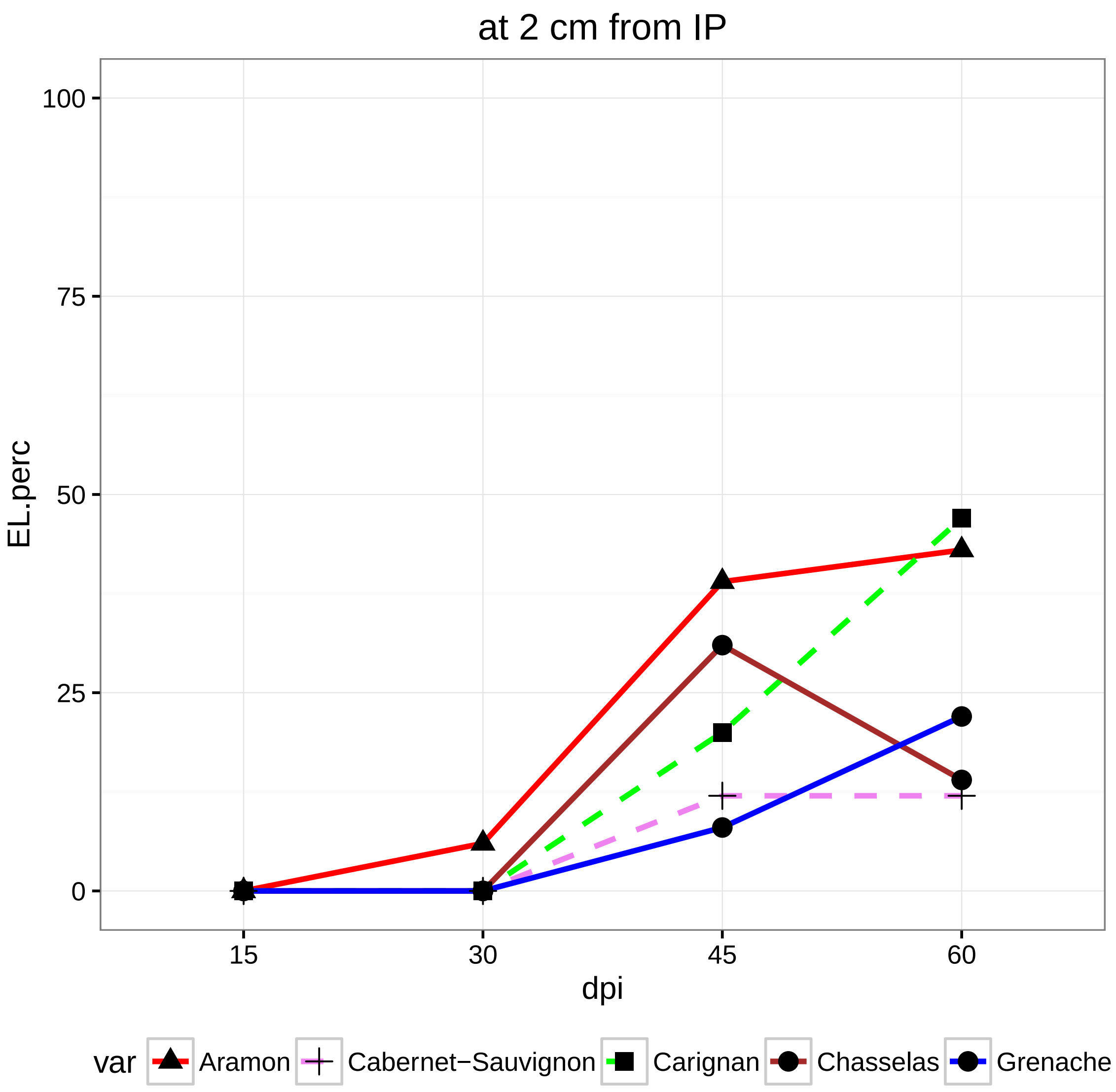

6 Experiment 3

qPCR as a tool to evaluate wood colonization by *E. lata*.

6.1 Figure 5

Plot raw data

```
# Function to calculate the standard error from dataframe x
  std.error <- function(x, na.rm = T) {
      sqrt(var(x, na.rm = na.rm)/length(x[complete.cases(x)]))
  }

# calculate means and std.error
  dpi.mean <- ddply(dpi.df, .(treat, PCR, dist_cm, dpi), summarise,
```

```
                    "nb_copies.mean"= mean(nb_copies, 2, na.rm = TRUE),
                    "nb_copies.std" = std.error(nb_copies, na.rm = TRUE),
                    count.no.NA = length(na.omit(nb_copies)))
  str(dpi.mean)
```

```
## 'data.frame':    34 obs. of  7 variables:
##  $ treat         : Factor w/ 3 levels "EL","H2O","T-": 1 1 1 1 1 1 1 1 1 1 ...
##  $ PCR           : Factor w/ 2 levels "actin","EL": 1 1 1 1 1 1 1 1 2 2 ...
##  $ dist_cm       : Factor w/ 2 levels "1-","1+": 1 1 1 1 2 2 2 2 1 1 ...
##  $ dpi           : int  15 30 45 60 15 30 45 60 15 30 ...
##  $ nb_copies.mean: num  18648 33384 34430 34367 14092 ...
##  $ nb_copies.std : num  6937 1114 1684 2015 775 ...
##  $ count.no.NA   : int  3 4 4 4 3 2 2 3 3 4 ...
```

```
  head(dpi.mean)
```

```
##   treat   PCR dist_cm dpi nb_copies.mean nb_copies.std count.no.NA
## 1    EL actin      1-  15          18648          6937           3
## 2    EL actin      1-  30          33384          1114           4
## 3    EL actin      1-  45          34430          1684           4
## 4    EL actin      1-  60          34367          2015           4
## 5    EL actin      1+  15          14092           775           3
## 6    EL actin      1+  30          17515           925           2
```

```
# remove actin PCR
  tmp <- subset(dpi.mean, PCR!="PAL" & PCR!="PCH" &
              treat!="H2O" & treat!="PAL" & treat!="PCH")
  levels(tmp$treat)[5]<- "CONTROLS"
  levels(tmp$treat)[1]<- "EL inoc"
  levels(tmp$PCR)[1] <- "Actin V.vinifera"
  levels(tmp$PCR)[2] <- "Beta-tubulin E.lata"
  levels(tmp$dist_cm) <- c("-1 cm below IP", "+1 cm above IP")

# colors
  my_col=c("grey","pink")

# Define the top and bottom of the errorbars
  limits <- aes(ymax= nb_copies.mean + nb_copies.std,
              ymin= nb_copies.mean - nb_copies.std)

# barplot and std.error
  p <- ggplot(tmp, aes(as.factor(dpi), nb_copies.mean, fill=PCR))
  #p <- p + ylim(0,40000)
  p <- p + geom_bar(stat="identity", position="dodge", color="black")
  p <- p + geom_errorbar(limits, position=position_dodge(.9),width=.2)
  p <- p + geom_text(aes(label=paste0(round(nb_copies.mean,0),"\n(n=",count.no.NA,")")),
                  position=position_dodge(width=0.9), vjust=-.1)
  p <- p + scale_fill_manual(values = my_col)
  p <- p + labs(title= "", x= "Number of days after inoculation",
              y= "Gene copy number (mean), by qPCR")
  p <- p + facet_grid(PCR ~ dist_cm*treat, scales = "free_y")
  p <- p + theme_bw() + theme(legend.position="top")
print(p)
```

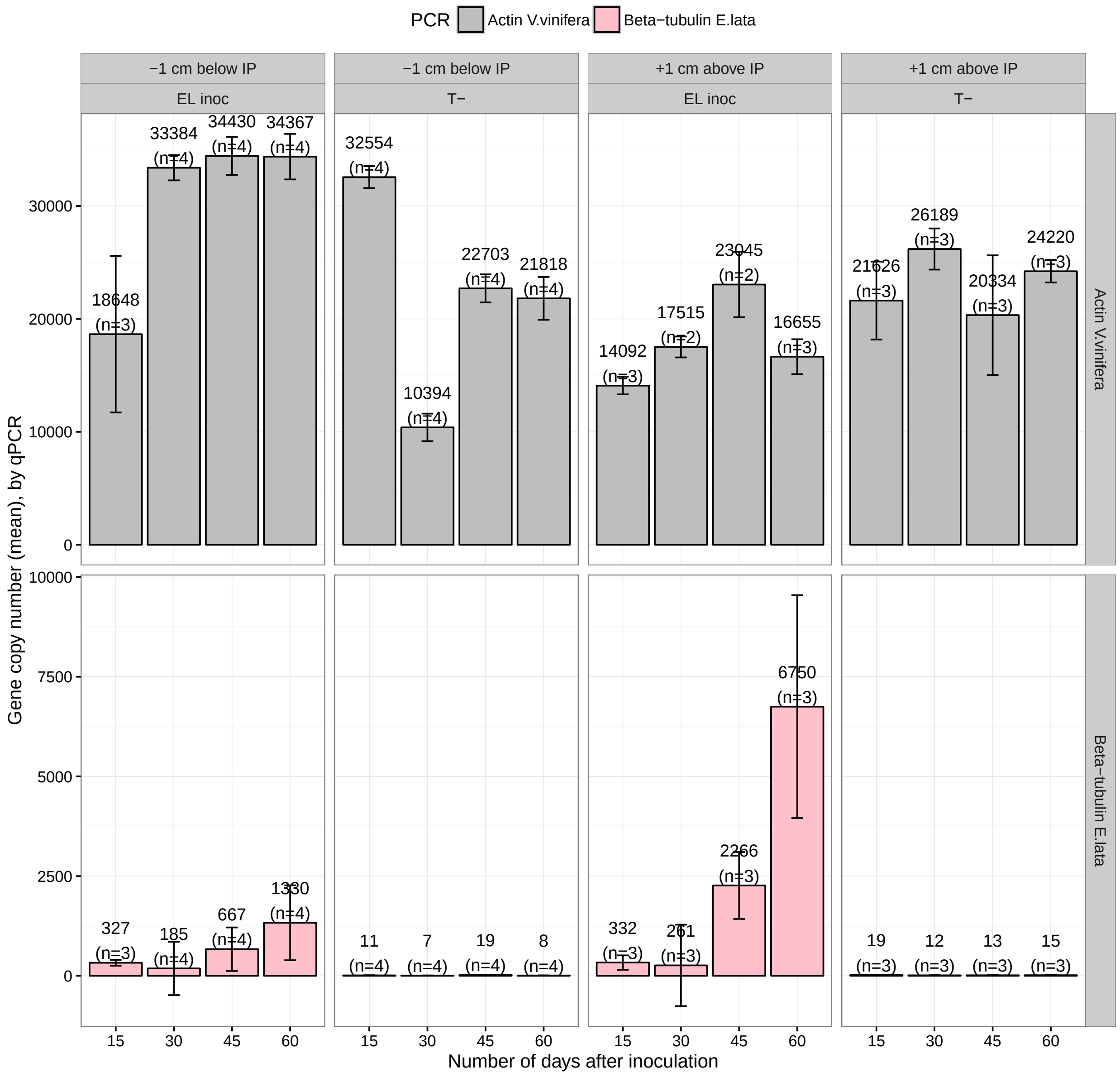

6.2 Figure S4

Wood colonization by *E. lata* monitored by qRT-PCR: ratio -tubulin / actin.

```
# format data before ratio
  d <- dpi.df[,c("sample","treat","PCR","dist_cm","dpi","nb_copies")]
  d$dpi <- factor(d$dpi)

# aggregate
  d2 <- cast(d, sample+treat+dist_cm+dpi ~ PCR, median)
```

```
## Using nb_copies as value column.  Use the value argument to cast to override this choice
```

```r
# calculate ratio
  d2$ratio <- d2$EL / d2$actin

# calculate RATIO means and std.error
  d2.mean <- ddply(d2, .(treat, dist_cm, dpi), summarise,
                   "ratio.mean"= mean(ratio, 2, na.rm = TRUE),
                   "ratio.std" = std.error(ratio, na.rm = TRUE),
                   "count" = length(na.omit(ratio)))

# Define the top and bottom of the errorbars
  limits <- aes(ymax= ratio.mean + ratio.std,
                ymin= ratio.mean - ratio.std)

# barplot
  p <- ggplot(na.omit(d2.mean), aes(as.factor(dpi), ratio.mean, fill=treat))
  p <- p + geom_bar(stat="identity", position="dodge", color="black")
  p <- p + ylim(0,0.75)
  p <- p + geom_errorbar(limits, position=position_dodge(.9),width=.2)
  p <- p + geom_text(aes(label=paste0(round(ratio.mean,2),"\n(n=",count,")")),
                     position=position_dodge(width=0.9), vjust=-.1)
  p <- p + scale_fill_manual(values = rep("lightblue",3))
  p <- p + labs(title= "", x= "Number of days after inoculation",
                y= "Ratio b-tubulin/actin")
  p <- p + facet_grid(. ~ dist_cm*treat, scales = "free_y")
  p <- p + theme_bw() + theme(legend.position="none")
print(p)
```

```
## Warning: Removed 1 rows containing missing values (geom_errorbar).
```

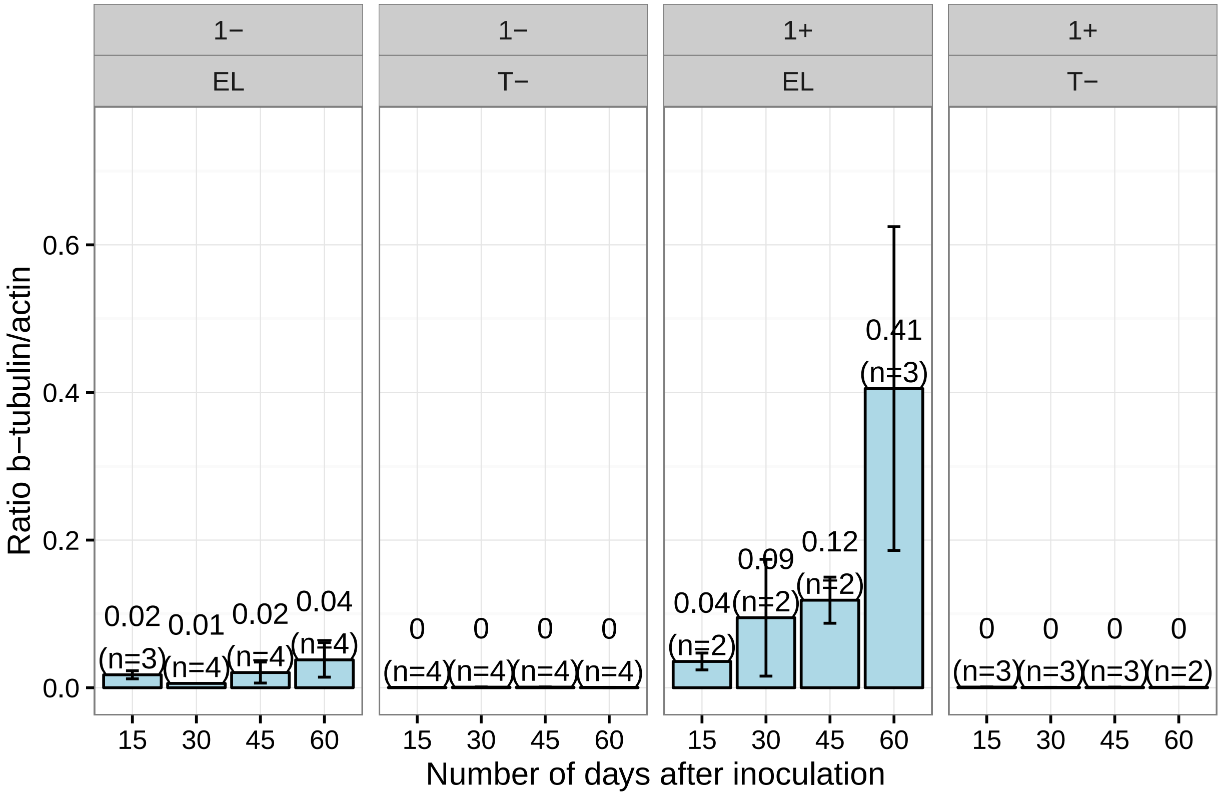

6.3 Analysis (figure 5)

6.3.1 Set up dataframe

```
# select data
  d <- dpi.df[,c("sample","treat","PCR","dist_cm","dpi","nb_copies")]
  d <- subset(d, treat!="T-" & treat!="H2O" & PCR!="actin")
  d <- droplevels(d)

# Convert variables to factor
  d <- within(d, {sample <- factor(sample)
                  treat <- factor(treat)
                  PCR <- factor(PCR)
                  dist_cm <- factor(dist_cm)
                  dpi <- factor(dpi)
              })
  str(d)
```

```
## 'data.frame':    32 obs. of  6 variables:
##  $ sample   : Factor w/ 32 levels "EL_2sem_1cm-_1",..: 1 2 3 4 9 10 11 12 17 18 ...
##  $ treat    : Factor w/ 1 level "EL": 1 1 1 1 1 1 1 1 1 1 ...
##  $ PCR      : Factor w/ 1 level "EL": 1 1 1 1 1 1 1 1 1 1 ...
##  $ dist_cm  : Factor w/ 2 levels "1-","1+": 1 1 1 1 1 1 1 1 1 1 ...
##  $ dpi      : Factor w/ 4 levels "15","30","45",..: 1 1 1 1 2 2 2 2 3 3 ...
##  $ nb_copies: num  327 NA 505 255 141 ...
```

6.3.2 Explore data (interaction plot)

```
# interaction plot for vizualization
  with(na.omit(d), interaction.plot(dpi, dist_cm, nb_copies,
                                    lty= c(1, 12), lwd = 3,
                                    xlab = "Days post-inoculation",
                                    ylab = "B-tubulin copy number",
                                    trace.label = "group"))
```

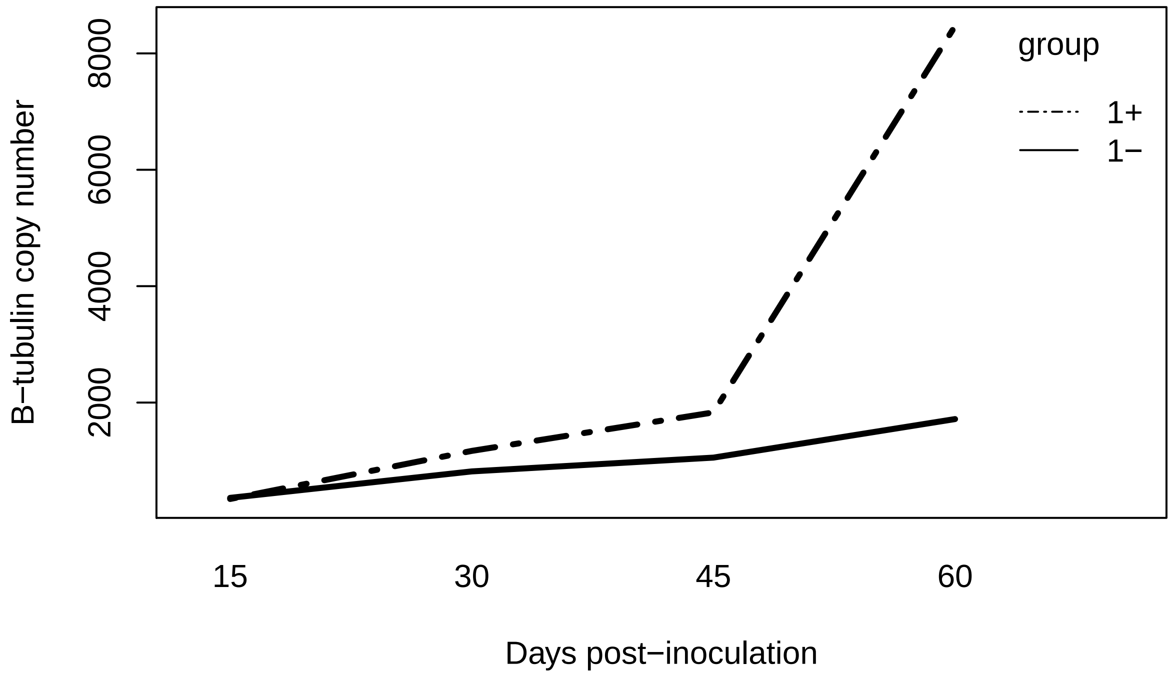

6.3.3 Linear model fit

Fit linear model (with repeated measures)

```
# estimate parameters of model
  reg.aov <- lm(nb_copies ~ dist_cm * dpi, data= d, na.action = na.exclude)
```

6.3.4 Diagnostics via residuals

```
# add standardized residuals (divided by sigma) to df
  d$res.std <- residuals(reg.aov) / summary(reg.aov)$sigma

# add fitted data to df
  d$fit.dat <- fitted(reg.aov)
```

```r
# plot diagnostics
  par(mfrow=c(1,2))
    # Plot standardized residuals versus fitted data
    plot(d$fit.dat, d$res.std,
         ylim=c(-5,5), main="Standardized residuals vs fitted data",
         xlab="Fitted data", ylab="Standardized residuals");
    abline(h=c(-2,0,2), lty=c(3,2,3), col=c("red", "black", "red"))

  # qqplot
    #qqnorm(d$res.std); abline(a=0, b=1, col="red")
     qqPlot(d$res.std)
```

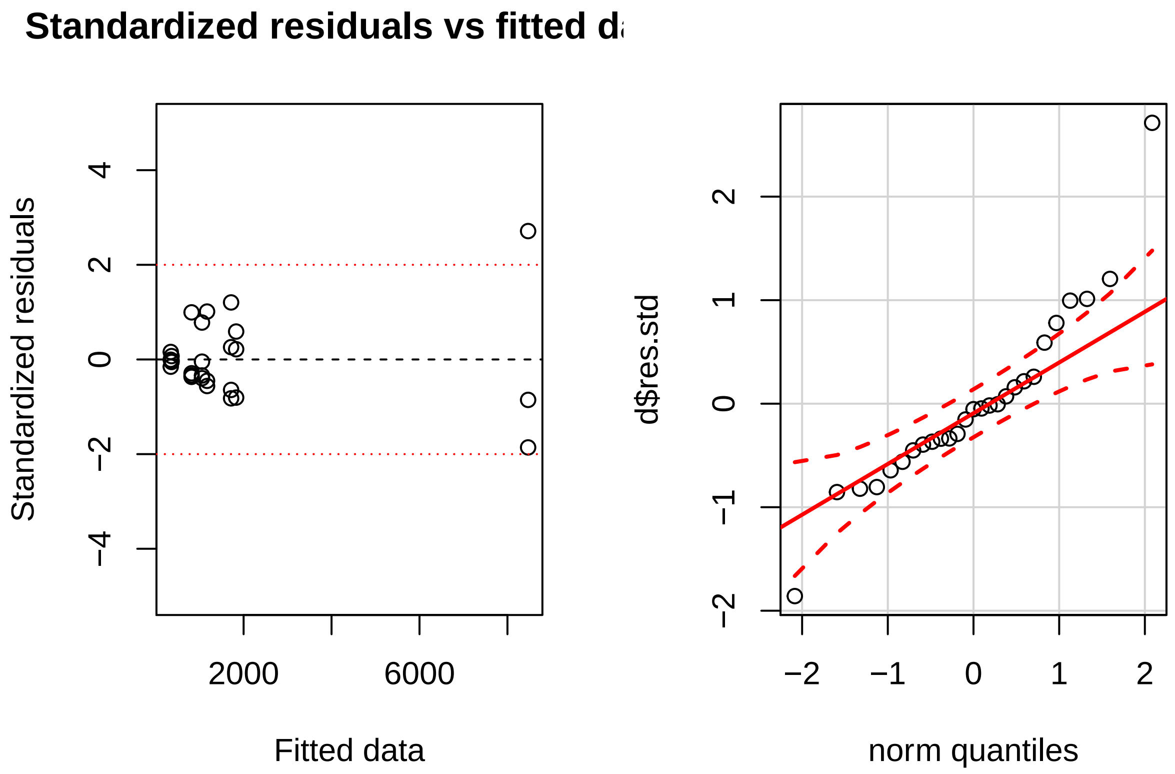


```r
  par(mfrow=c(1,1))

# other diagnostics
  # plot(reg.aov)
```

In theory, 95% of residuals should roughly be in the interval [-2,2]. Here we observed one outlier, but all other residuals are roughly in the 95% interval. We therefore decided to carry on with the analysis of variance.

6.3.5 Anova

Analysis of variance for inoculated samples collected at 1 cm from IP (above and below).

```r
# analysis of variance
  summary(reg.aov)
```

```
##
## Call:
## lm(formula = nb_copies ~ dist_cm * dpi, data = d, na.action = na.exclude)
##
## Residuals:
##    Min     1Q Median     3Q    Max
##  -3743   -852   -107    479   5463
##
## Coefficients:
##                  Estimate Std. Error t value Pr(>|t|)
## (Intercept)           362       1163    0.31   0.7587
## dist_cm1+             -19       1644   -0.01   0.9909
## dpi30                 455       1538    0.30   0.7707
## dpi45                 692       1538    0.45   0.6580
## dpi60                1354       1538    0.88   0.3898
## dist_cm1+:dpi30       371       2252    0.16   0.8707
## dist_cm1+:dpi45       796       2252    0.35   0.7277
## dist_cm1+:dpi60      6773       2252    3.01   0.0072 **
## ---
## Signif. codes:  0 '***' 0.001 '**' 0.01 '*' 0.05 '.' 0.1 ' ' 1
##
## Residual standard error: 2010 on 19 degrees of freedom
##   (5 observations deleted due to missingness)
## Multiple R-squared:  0.665,  Adjusted R-squared:  0.542
## F-statistic:  5.4 on 7 and 19 DF,  p-value: 0.00158
```

```
anova(reg.aov)
```

```
## Analysis of Variance Table
##
## Response: nb_copies
##             Df   Sum Sq  Mean Sq F value Pr(>F)
## dist_cm      1 24687059 24687059    6.09 0.0233 *
## dpi          3 76397979 25465993    6.28 0.0038 **
## dist_cm:dpi  3 52071749 17357250    4.28 0.0181 *
## Residuals   19 77049731  4055249
## ---
## Signif. codes:  0 '***' 0.001 '**' 0.01 '*' 0.05 '.' 0.1 ' ' 1
```

7 Experiment 4

Comparison of *E. lata* aggressiveness by qPCR.

7.1 Figure 6

Select and plot qPCR data

```
# select data
  d <- d.agr

# Set marging
  par(mar = c(7, 4, 5, 1))
```

```r
# Boxplot tubulin vs treatment
  boxplot(d$btub_Elata ~ d$Treatment,
          main=paste0("Comparison of E.lata isolates \nfor wood colonization"),
          ylab="Genome copy number", xlab="E. lata isolates",
          varwidth= TRUE, notch= FALSE, las= 2, col= c("brown", "green"))

# Add points
  points(d$btub_Elata ~ d$Treatment, pch= 19)
```

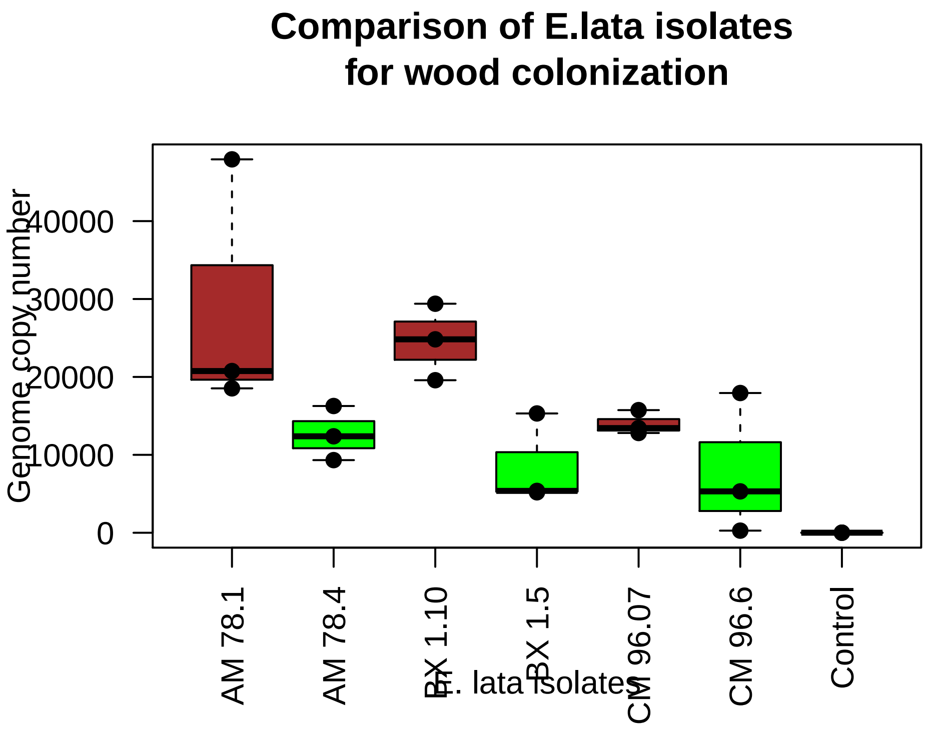

7.2 Analysis (figure 6)

7.2.1 Set up dataframe

```r
# select data without controls
  d <- droplevels(subset(d.agr, Treatment != "Control"))
  d$sample <- factor(d$sample)
  str(d)
```

```
## 'data.frame':    18 obs. of  7 variables:
##  $ sample    : Factor w/ 18 levels "1","2","3","4",..: 1 2 3 4 5 6 7 8 9 10 ...
##  $ Treatment : Factor w/ 6 levels "AM 78.1","AM 78.4",..: 1 1 1 2 2 2 3 3 3 4 ...
##  $ Rep       : int  1 2 3 1 2 3 1 2 3 1 ...
##  $ btub_Elata: num  18533 47926 20755 9323 12379 ...
##  $ sd        : num  605 727 1519 199 1498 ...
##  $ agr_level : Factor w/ 2 levels "high-aggressive",..: 1 1 1 2 2 2 1 1 1 2 ...
```

```
##  $ stroma    : Factor w/ 3 levels "AM","BX","CM": 1 1 1 1 1 1 2 2 2 2 ...
```

7.2.2 Explore data (interaction plot)

```
# interaction plot for vizualization
  with(na.omit(d), interaction.plot(agr_level, stroma, btub_Elata,
                                    lty= c(1, 9, 12), lwd = 3,
                                    xlab = "Aggressiveness",
                                    ylab = "B-tubulin copy number",
                                    trace.label = "group"))
```

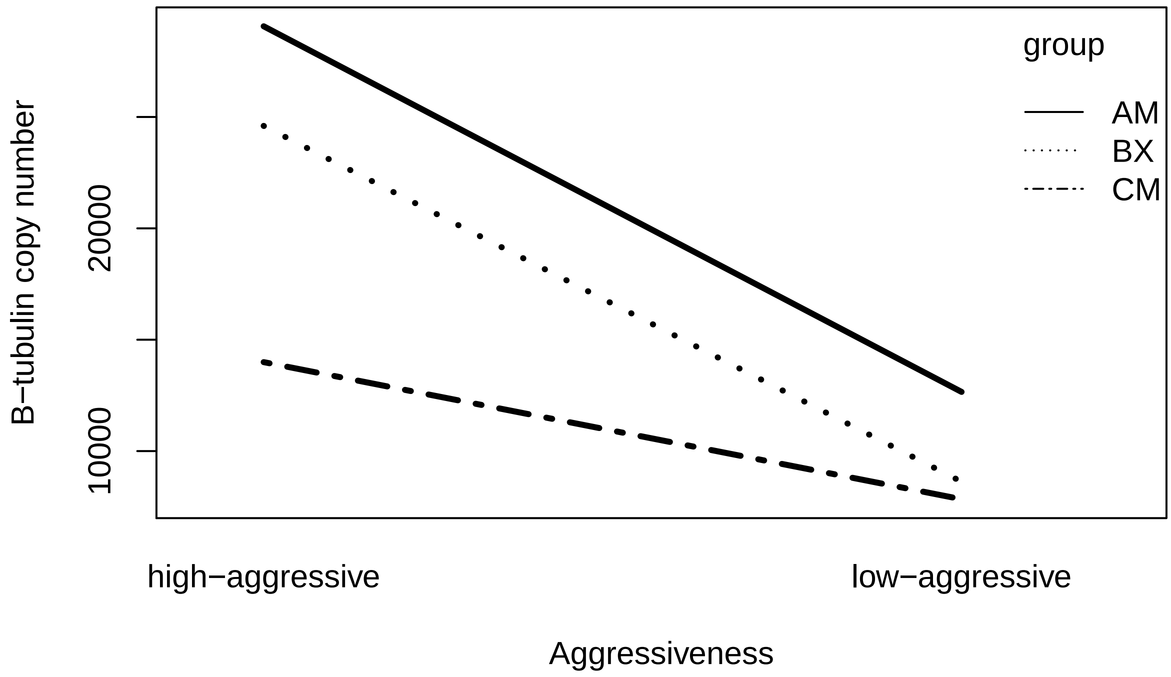

7.2.3 Linear model fit

Fit linear model

```
# estimate parameters of model
  reg.aov <- lm(btub_Elata ~ stroma * agr_level, data= d)
```

7.2.4 Diagnostics via residuals

```
# add standardized residuals (divided by sigma) to df
  d$res.std <- residuals(reg.aov) / summary(reg.aov)$sigma

# add fitted data to df
```

```r
  d$fit.dat <- fitted(reg.aov)

# plot diagnostics
  par(mfrow=c(1,2))
    # Plot standardized residuals versus fitted data
    plot(d$fit.dat, d$res.std,
         ylim=c(-5,5), main="Standardized residuals vs fitted data",
         xlab="Fitted data", ylab="Standardized residuals");
    abline(h=c(-2,0,2), lty=c(3,2,3), col=c("red", "black", "red"))

# qqplot
    #qqnorm(d$res.std); abline(a=0, b=1, col="red")
    qqPlot(d$res.std)
```

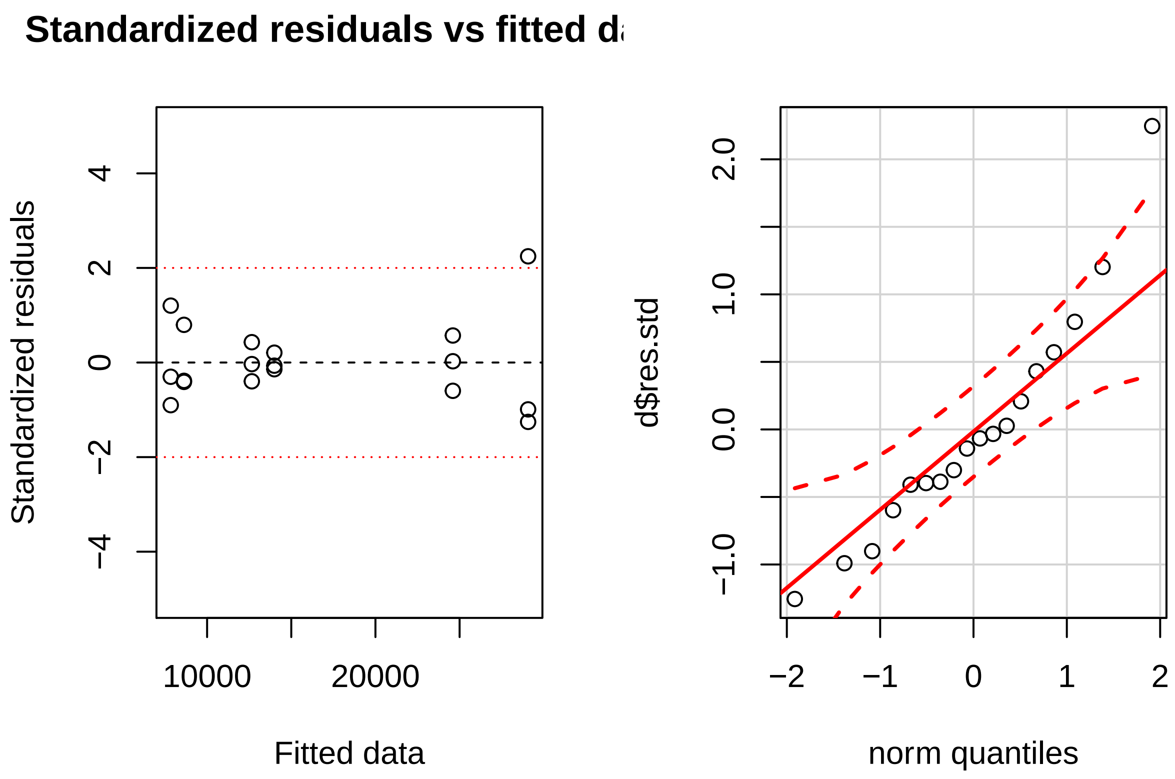


```r
  par(mfrow=c(1,1))
```

In theory, 95% of residuals should roughly be in the interval [-2,2]. Here we observed one outlier, but all other residuals are roughly in the 95% interval. We therefore decided to carry on with the analysis of variance.

7.2.5 Anova

Analysis of variance for inoculations performed with different isolates from three stroma origins.

```r
# analysis of variance
  summary(reg.aov)
```

```
##
```

```
## Call:
## lm(formula = btub_Elata ~ stroma * agr_level, data = d)
##
## Residuals:
##    Min     1Q Median     3Q    Max
## -10538  -3410   -873   3145  18855
##
## Coefficients:
##                                  Estimate Std. Error t value Pr(>|t|)
## (Intercept)                         29071       4847    6.00  6.2e-05 ***
## stromaBX                            -4471       6854   -0.65    0.526
## stromaCM                           -15077       6854   -2.20    0.048 *
## agr_levellow-aggressive            -16415       6854   -2.39    0.034 *
## stromaBX:agr_levellow-aggressive      441       9693    0.05    0.964
## stromaCM:agr_levellow-aggressive    10260       9693    1.06    0.311
## ---
## Signif. codes:  0 '***' 0.001 '**' 0.01 '*' 0.05 '.' 0.1 ' ' 1
##
## Residual standard error: 8390 on 12 degrees of freedom
## Multiple R-squared:  0.575,  Adjusted R-squared:  0.397
## F-statistic: 3.24 on 5 and 12 DF,  p-value: 0.0441
```

```
anova(reg.aov)
```

```
## Analysis of Variance Table
##
## Response: btub_Elata
##                  Df   Sum Sq  Mean Sq F value Pr(>F)
## stroma            2 2.99e+08 1.49e+08    2.12  0.163
## agr_level         1 7.43e+08 7.43e+08   10.54  0.007 **
## stroma:agr_level  2 1.01e+08 5.05e+07    0.72  0.508
## Residuals        12 8.46e+08 7.05e+07
## ---
## Signif. codes:  0 '***' 0.001 '**' 0.01 '*' 0.05 '.' 0.1 ' ' 1
```
